# Spatiotemporal expression and coexpression patterns of SRPK1 in the human brain: A neurodevelopmental perspective

**DOI:** 10.1002/brb3.3341

**Published:** 2023-12-31

**Authors:** Jing‐jing Wang, Sagor Kumar Roy, Yu‐ming Xu

**Affiliations:** ^1^ Department of Neurology The First Affiliated Hospital of Zhengzhou University Zhengzhou University Zhengzhou Henan P. R. China

**Keywords:** BrainSpan Atlas of the Developing Human Brain, coexpression, neurodevelopment, spatiotemporal expression patterns, srpk1

## Abstract

**Background:**

SRPK1 is a splicing‐related protein that plays an important role in the development and function of the human brain. This article presents evidence that SRPK1 has distinct spatiotemporal expression patterns enriched in processes related to neurodevelopmental disorders across development.

**Material and method:**

We used the BrainSpan growing mammalian brain transcriptome to evaluate the distribution of SRPK1 throughout the entire brain. RNA‐sequencing data were gathered from 524 tissue samples recovered from 41 postmortem brains of physiologically normal individuals spanning early developing fetus (8 postconception weeks, PCW) to later life (40 years of age). Using the Allen Human Brain Atlas (AHBA) dataset, we analyzed the spatial gene expression of 15 adult human brains. Using Toppgene, we identified genes that exhibit significant coexpression with SRPK1.

**Results:**

We found evidence that analyzing the spatiotemporal gene expression profile and identifying coexpressed genes reveals that SRPK1 expression is involved in various neurodevelopmental and somatic events throughout the lifetime.

**Conclusion:**

Our findings highlight the importance of detailed maps of gene expression in the human brain for improved human‐to‐human translation and illustrate differences in SRPK1 expression across anatomical areas and developmental stages in healthy human brain tissue.

## Graphical abstract

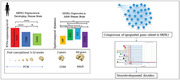

## 1 INTRODUCTION

The serine‐arginine protein kinase (SRPK) group includes three members: SRPK1, SRPK2, and SRPK3. Although each member of the SRPK subfamily has a distinct expression pattern, these proteins are conserved kinases with ∼70% amino acid identity (Chan & Ye, 2013). The SRPK subfamily is part of a conserved family of serine/threonine kinases that phosphorylate the serine residues found in SR/RS dipeptide patterns; this family has over 50 members identified in the genomes of mammals, nematodes, insects, fungi, and plants (Giannakouros et al., 2011). SRPK1 has served as the foundation for most research on human SRPKs to date.

Various physiological functions, including mRNA maturation, nuclear import, chromatin structure, and progenitor cell growth, have been linked to the regulation of SRPK1. Among its substrates are SR splicing factors, which play a crucial role in intrinsic and alternative splicing; these factors are involved in various stages of mRNA expression, such as transcriptional elongation and protein synthesis (Mytilinaios et al., 2012). SRPK1 is a protein kinase that phosphorylates proteins with serine‐arginine‐rich (SR) domains that are essential for modulating multiple RNA‐processing mechanisms, such as translation, alternative splicing, and RNA stability (Long & Caceres, 2009). Increased expression of SRPK1 and its downstream targets is observed in numerous types of cancer, including colon, breast, pancreatic, and prostate cancer, as well as in numerous physiological and pathological processes (Bullock & Oltean, 2017). Alternative splicing enables significant molecular variety in the human nervous system, allowing hundreds of unique proteins to be produced from far fewer genes (Graveley, 2001). Protein isoforms specific to neurons are crucial for neuronal growth, memory, learning, and cell communication. Because of alternative splicing, neurotransmitter receptors maintain significant diversity, preserving the uniqueness of neural response connections (Grabowski & Black, 2001). When a cell undergoes osmotic shock in response to stress events, SRPK1 splits from the intracellular cochaperone complex and enters the nucleus, where it phosphorylates SR proteins to modify RNA splicing (Zhong et al., 2009). Previous research has demonstrated that SRPK1 controls the alternative splicing of exon 10, which is related to the etiology of tauopathies such as frontotemporal dementia and Alzheimer's disease (AD) (Hartmann et al., 2001). Therefore, SRPK1 may play a vital role in controlling the production of protein isoforms specific to neurons, including various neurotransmitter receptor subtypes. Previous studies have demonstrated that SRPK1 is expressed in the central nervous system (CNS) of mammals (Erdö et al., 2004).

Knowledge of the brain functions affected and unaffected by SRPK1 could be crucial for understanding disease involvement because the participation of specific proteins in disease is frequently poorly preserved across species. In this study, we intend to utilize the BrainSpan growing mammalian brain transcriptome to evaluate the distribution of SRPK1 throughout the brain (https://www.brainspan.org/rnaseq/). The Allen Human Brain Atlas is intended to provide a complete map of transcript utilization throughout the adult human brain. The atlas provides complete anatomical coverage with sufficiently high quality to capture cell nuclei; all data are from DNA microarrays of clinically normal brains profiled by quantitative gene‐level transcriptomics. Moreover, anatomical brain imaging data were collected from each individual to enable correlations between imaging and transcriptomic information and to show gene expression data in their original three‐dimensional structural coordinate space. The files are freely accessible through the Allen Brain Atlas data gateway (http://www.brain‐map.org).

## 2 MATERIALS AND METHODS

### 2.1 BrainSpan Developing Human Brain transcriptome

The BrainSpan Atlas of the Developing Human Brain transcriptome was used to download RNA‐sequencing‐derived data on SRPK1 expression (Miller et al., 2014). RNA sequencing (RNA‐seq) data were gathered from 524 tissue samples recovered from 41 postmortem brains of physiologically normal individuals from early fetal development (8 postconceptional weeks, PCW) to later life (40 years of age) (Table S1). We enrolled from 1st week to 37 weeks for PCW, childhood from after 1 month to 18 years and adulthood start from more than 18 years and afterwards. The samples were collected via macroscopic section from 8 to 16 areas per brain. The brain was subdivided into 19 nonoverlapping anatomical regions as follows: A1C: primary auditory cortex (core); AMY: amygdaloid complex; CB: cerebellum; CBC: cerebellar cortex; CGE: caudal ganglionic eminence; DFC: dorsolateral prefrontal cortex; DTH: dorsal thalamus; HIP: hippocampus; IPC: posteroventral (inferior) parietal cortex; ITC: inferolateral temporal cortex; M1C: primary motor cortex; M1C‐S1C: primary motor and sensory cortices; MD: mediodorsal nucleus of the thalamus; MFC: anterior (rostral) cingulate cortex and medial prefrontal cortex; OFC: orbitofrontal cortex; PCx: parietal neocortex; S1C: primary somatosensory cortex; STC: posterior (caudal) superior temporal cortex; STR: striatum; V1C: primary visual cortex; VFC: ventrolateral prefrontal cortex.

The sequencing data were coregistered with regional brain MRI scans in the Montreal Neurological Institute (MNI) coordinate space. The microarray‐based data formed the basis for a freely navigable online atlas containing readers for histology, sample location, MRI, and three‐dimensional (3D) gene expression visualization. Using a high throughput in situ hybridization (ISH) platform, large‐scale ISH datasets are being generated to complement and validate microarray data for many targets (M. J. Hawrylycz et al., 2012). The BrainSpan website (http://www.brainspan.org/rnaseq/search/index.html) provides information about tissue collection and data processing. The RNA‐seq data were annotated with gene and exon information from GENCODE version 10 (GRCh37–Ensembl 65). Exon expression levels were quantified in RPKM (reads per kilobase per million mapped reads) units.

### 2.2 Adult human brain expression data

To better understand the function of SRPK1, we consulted the Allen Human Brain Atlas (AHBA) dataset (https://human.brain‐map.org), which includes information on the spatial gene expression of fifteen adult human brains (M. J. Hawrylycz et al., 2012). Postmortem brain samples were retrieved from 11 males and 4 females aged 24 to 55 years (mean age: 40 years) with no known psychological disorders either by manual macroscopic dissection (cortical and specific subcortical structures) or by beam microscopic dissection (subcortical and brainstem areas). RNA was collected from 363 to 946 different samples of each brain (1014 samples in total) and analyzed on experimental custom microarrays, which included the 444K Agilent Whole Human Genome probes and an additional 16,000 custom probes. For loci with two probes, the probe with the greatest variance was chosen. For genes with three or more probes, the gateway of each probe was assessed (sum of Pearson correlations with all other tests, determined for each brain and then averaged), and the probe with the most substantial connection was chosen. The expression data of 19,991 genes were normalized within each brain using the *z* scores, and SRPK1 gene expression *z* score values were mapped to AHBA anatomical pictures for display (M. J. Hawrylycz et al., 2012).

### 2.3 Coexpression analysis

We assessed the spatial correlation (using Pearson's correlation coefficient) among all genes in the AHBA (19,991 genes) to determine the functional link between the SRPK1 gene and all other genes in the adolescent human brain by integrating all data from six donors (3902 samples). We assessed the spatiotemporal correlation (Pearson's) among all exons (241,690 exons) in the BrainSpan dataset to determine their functional relationships with SRPK1 during development. Based on the relationships, genes were ranked in descending order. To develop a ranked gene list, the rank of each gene's most highly correlated exon was assigned to each gene (https://genefriends.org). A functional annotation analysis was conducted on the top 1% of gene sets (strongest positive correlations). Using ToppGene, a functional annotation analysis was performed (https://toppgene.cchmc.org/) (Chen et al., 2009). Human Phenotype, Gene Ontology (GO) Biological Process, GO Molecular Function, GO Cellular Component, and Pathway terms enriched at a false discovery rate (FDR)‐corrected *q*‐value of .05 were included.

## 3 RESULTS

### 3.1 SRPK1 expression throughout the developing brain

**FIGURE 1:**
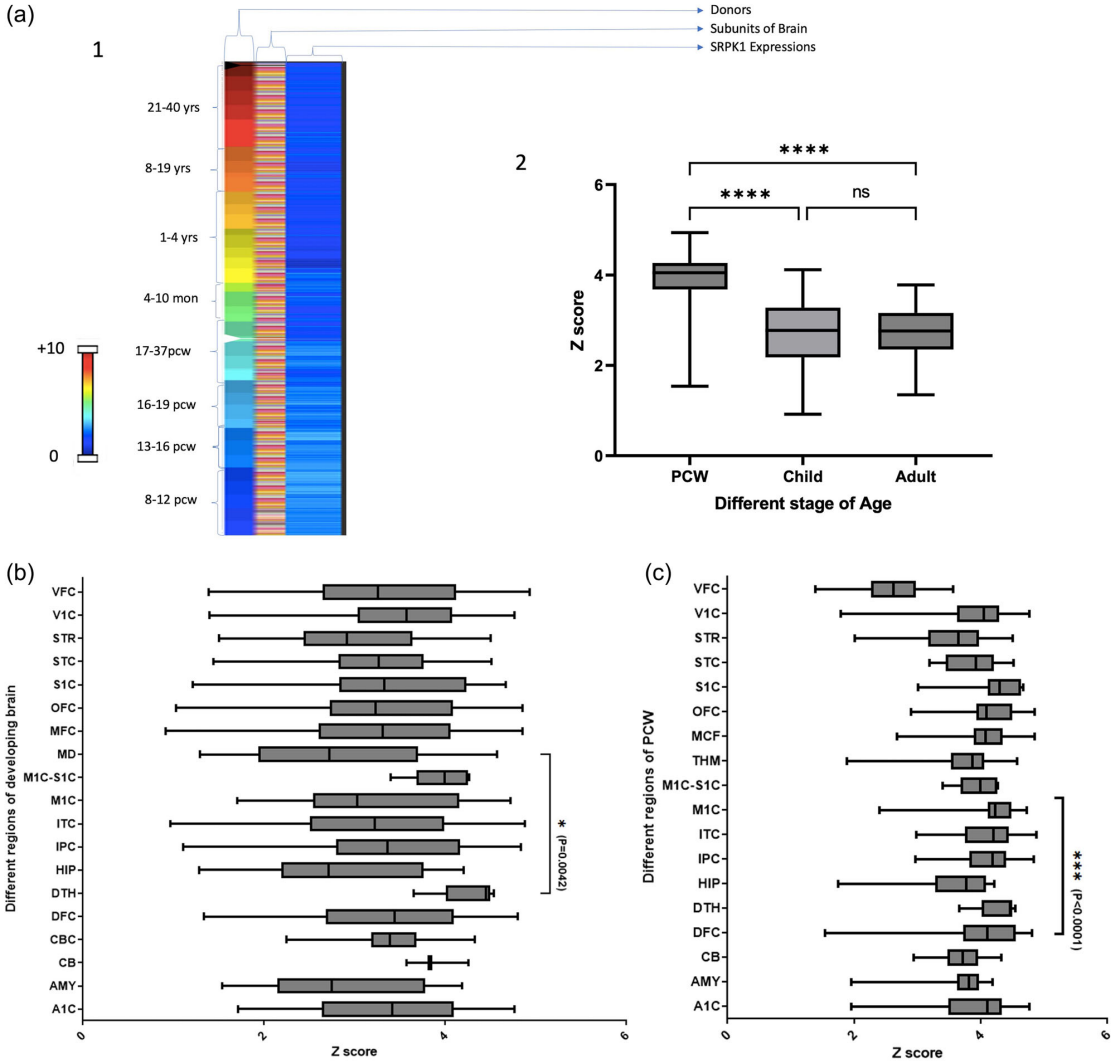
(a) (1) Transcriptomic data on SPRK1 from the BrainSpan Atlas of the Developing Human Brain shown as a heatmap of donor age and RNA‐seq‐measured levels of SRPK1 expression in different structures within the brain (https://www.brainspan.org/rnaseq/). (2) RNA‐seq‐measured levels of SRPK1 expression in the human brain is highest prenatally, with significantly higher levels during the early PCW than during childhood and adulthood (*p* value < .0001) and marginally higher levels during childhood than during adulthood (no significant difference) according to one‐way ANOVA. (b) As measured by RNA‐seq, the highest levels of SRPK1 expression (*z* scores) were found in the posteroventral (inferior) parietal cortex, inferolateral temporal cortex, primary motor cortex and dorsal thalamus (*p* value = .0042 by one‐way ANOVA). A1C: primary auditory cortex (core), AMY: amygdaloid complex, CB: cerebellum, CBC: cerebellar cortex, CGE: caudal ganglionic eminence, DFC: dorsolateral prefrontal cortex, DTH: dorsal thalamus, HIP: hippocampus, IPC: posteroventral (inferior) parietal cortex, ITC: inferolateral temporal cortex, M1C: primary motor cortex, M1C‐S1C: primary motor‐sensory cortex, MD: mediodorsal nucleus of thalamus, MFC: anterior (rostral) cingulate (medial prefrontal) cortex, OFC: orbital frontal cortex, PCx: parietal neocortex, S1C: primary somatosensory cortex, STC: posterior (caudal) superior temporal cortex STR: striatum, V1C: primary visual cortex, VFC: ventrolateral prefrontal cortex. (c) Increased RNA‐seq‐measured SRPK1 expression in the early PCW was found throughout the posteroventral (inferior) parietal cortex, inferolateral temporal cortex, primary motor cortex, and dorsal thalamus, with *p* values < .0001 by one‐way ANOVA.

The expression of SRPK1 was evaluated during development using the transcriptome data from the BrainSpan Atlas of the Developing Human Brain. The BrainSpan atlas provides RNA‐seq expression analyses of 16 distinct brain regions from 41 donor brains from early fetal development (8 PCW) to adulthood (40 years). Our analysis showed that SRPK1 expression increases throughout the human brain immediately before birth, with significantly higher levels during the early PCW than during childhood and adulthood (*p* value < .0001) and marginally higher levels during childhood than during adulthood (no significant difference) according to one‐way ANOVA using Prism 9.5 software (Figure 1a; Table S1).

In the whole developing brain, most SRPK1 expression was found in the posteroventral (inferior) parietal cortex, inferolateral temporal cortex, primary motor cortex and dorsal thalamus, with a *p* value of .0042 by one‐way ANOVA using Prism 9.5 software (Figure 1b). The brain was subdivided into 19 nonoverlapping anatomical regions as follows: A1C: primary auditory cortex (core); AMY: amygdaloid complex; CB: cerebellum; CBC: cerebellar cortex; CGE: caudal ganglionic eminence; DFC: dorsolateral prefrontal cortex; DTH: dorsal thalamus; HIP: hippocampus; IPC: posteroventral (inferior) parietal cortex; ITC: inferolateral temporal cortex; M1C: primary motor cortex; M1C‐S1C: primary motor and sensory cortices; MD: mediodorsal nucleus of the thalamus; MFC: anterior (rostral) cingulate cortex and medial prefrontal cortex; OFC: orbitofrontal cortex; PCx: parietal neocortex; S1C: primary somatosensory cortex; STC: posterior (caudal) superior temporal cortex; STR: striatum; V1C: primary visual cortex; VFC: ventrolateral prefrontal cortex.

Previously, an increase in RNA‐seq‐measured SRPK1 expression was observed during the early PCW relative to the average over the lifespan. During this stage of development, increased SRPK1 expression was also found in the posteroventral (inferior) parietal cortex, inferolateral temporal cortex, primary motor cortex, and dorsal thalamus by one‐way ANOVA using Prism 9.5 software (Figure 1c).

During childhood, we observed increased SRPK1 expression in the primary visual cortex (striate cortex, region V1/17), cerebellar cortex and posteroventral (inferior) parietal cortex. Adults exhibited elevated SRPK1 expression throughout the cerebellum and primary visual cortex (striate cortex, region V1/17) and increased divergence within brain areas, reflecting the trends outlined previously (see Table S1).

### 3.2 Spatial pattern of adult brain SRPK1 expression

**FIGURE 2:**
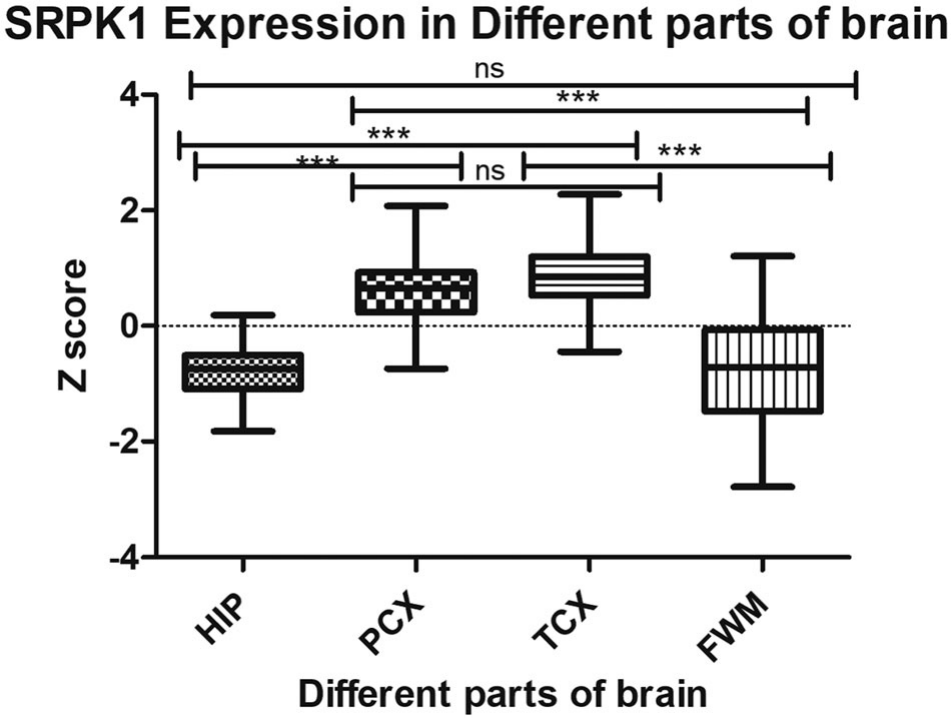
As measured by RNA‐seq in the AHBA (https://human.brain‐map.org), SRPK1 expression is elevated in the temporal cortex (TCX) of the adult brain (*p* value < .0001, one‐way ANOVA).

We analyzed the spatial patterns of SRPK1 gene expression throughout the adult brain using the AHBA, which has far greater accuracy than the BrainSpan atlas but lacks a temporal component. The AHBA provides microarray gene expression profiles from hundreds of samples obtained from six adult human brains, providing a comprehensive examination of the local expression of genes in the human brain. However, there is no way to discriminate between different isoforms because the sample preparation method used oligo‐dT primers, which acquire only the end of the gene. We found that SRPK1 expression was elevated in the temporal cortex (TCX) of the adult brain (*p* value < .0001, one‐way ANOVA using Prism 9.5 software) as well as in the parietal neocortex (PTX) in comparison to the mean expression levels of the fifteen donors. The hippocampus (HIP) and forebrain white matter (FWM) expression had relatively low expression compared to the PCX and TCX (Figure 2; see Table S2).

### 3.3 The functional significance of spatiotemporal coexpression with SRPK1

We assessed the links between the temporal and spatial expression patterns of the SRPK1 gene and those of other genes to obtain a more profound understanding of the functional significance of the SRPK1 gene throughout the development of the human brain and its relationship with neurodevelopmental diseases.

Coexpression analysis is a widely used method that uses large‐scale expression data and the “guilt by association” principle to infer functional relationships between genes (Stuart et al., 2003). The most significant spatiotemporal relationships were identified between a gene set derived from a study with 62 annotated datasets and a GWAS‐derived gene set (www.gwascentral.org) enriched in AD (*p* < .05). Genome‐wide association research was conducted using 469,438 single nucleotide polymorphisms (SNPs) linked to AD risk and age at AD onset.

**FIGURE 3:**
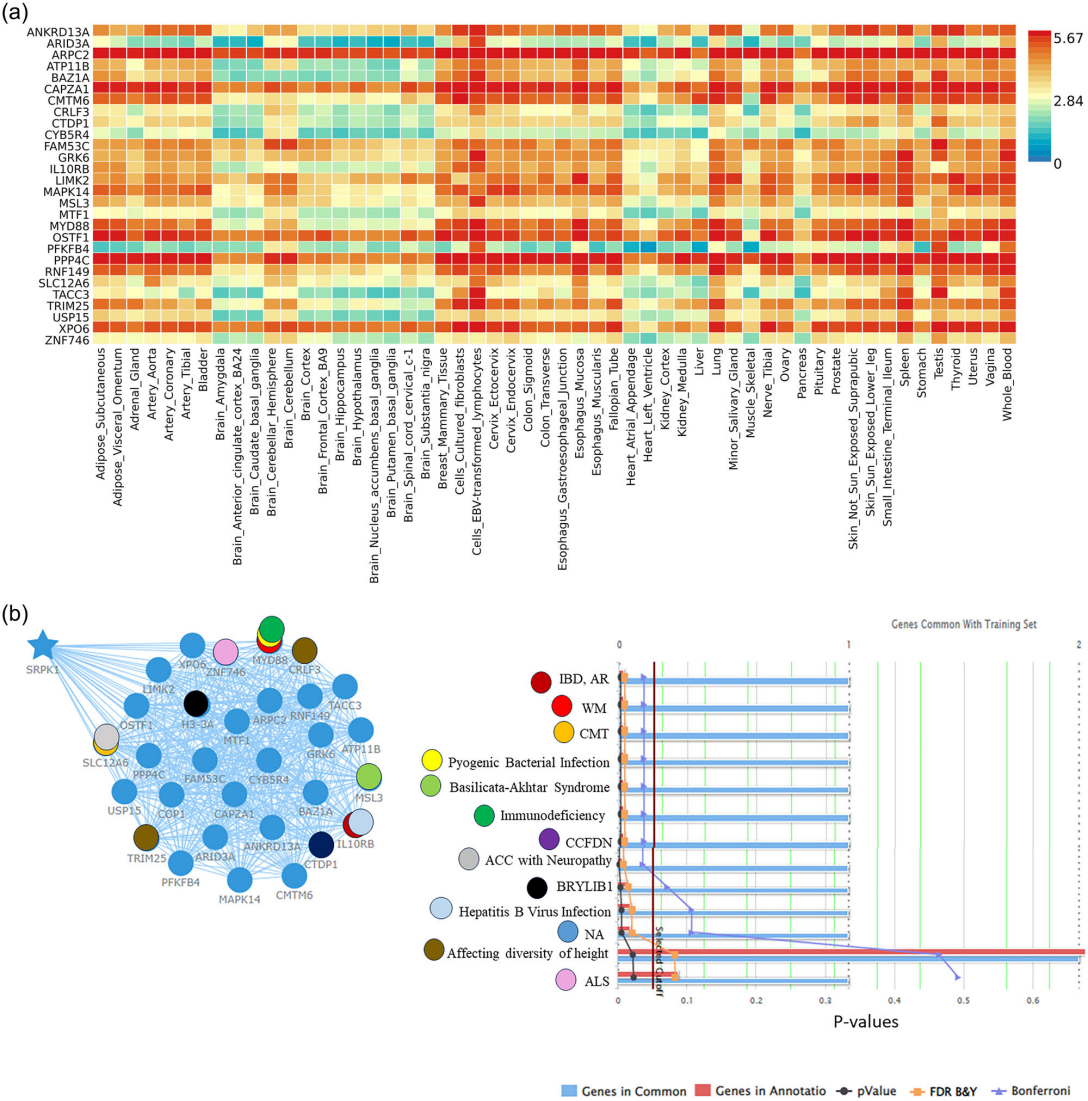
(a) Top 28 genes that have significant coexpression with SRPK1. Functional Mapping and Annotation of Genome‐Wide Association Studies (FUMA)was used to identify upregulated genes with differential expression in cerebellar tissue and other tissues (*p* < .0001) (https://fuma.ctglab.nl/). (b) Using ToppGene to evaluate genes that exhibit substantial coexpression with SRPK1 for relevance in gene. The phenotype associations were found gait abnormalities, neurodevelopmental delay IBD, AR = Inflammatory bowel disease, autosomal recessive, Waldenstrom macroglobulinemia (WM), Charcot‐Marie‐Tooth disease (CMT), MSL3 (Basilicata‐Akhtar) Syndrome, Congenital Cataracts Facial Dysmorphism Neuropathy (CCFDN), Agenesis of the corpus callosum (ACC) with Neuropathy, Bryant‐Li‐Bhoj neurodevelopmental syndrome‐1 (BRYLIB1), Nothing abnormalities (NA), affecting diversity of adult human height, Amyotrophic lateral sclerosis (ALS), agenesis of corpus callosum related peripheral neuropathy using Toppgene. (https://toppgene.cchmc.org/).

Three of the top 120 SNPs by *p* value in the collected data and one of the Cox analyses of the Canadian dataset showed substantial evidence for connection at *p* < .05 by multivariable equation analysis of the United Kingdom Medical Research Council dataset; these three polymorphisms were rs7019241 (GOLPH2), rs10868366 (GOLPH2), rs9886784 (chromosome 9), and rs10519262 (intergenic between ATP8B4 and SLC27A2). Again, this genome‐wide association analysis identified the APOE association disequilibrium area as a significant risk indicator for AD. The association with this area could result from the coevolution of several susceptibility alleles, such as APOC1. Due solely to linkage disequilibrium with APOE, the SNP rs4420638 was substantially linked to AD among patients with the same APOC1 genotype, although unadjusted. Additionally, we share new data supporting other potential factors of genetic vulnerability to AD that can be investigated in future research. Furthermore, there was an enrichment of genes related to Parkinson's disease (*p* < .01) in an amyotrophic lateral sclerosis GWAS (*p* < .0001). Using the GTEx database (version 8), we assessed the density of this gene set in 54 tissue types throughout the body (Carithers & Moore, 2015; “Human genomics. The Genotype‐Tissue Expression (GTEx) pilot analysis: multitissue gene regulation in humans,” 2015). From the GTEx 8 version expressed in many tissues, 30 genes expression patterns dataset was gathered from https://genefriends.org. We found the significant 28 coexpression genes of SRPK1 (Table S3) and the differential expression mostly in cerebellum tissues and other tissues using “Functional Mapping and Annotation of Genome‐Wide Association Studies” (FUMA) (*p* < .0001, Bonferroni‐adjusted; Figure 3A; from https://fuma.ctglab.nl/; Table S4). However, COP1 and H3‐3A genes couldn't gather in FUMA for observing their expression level in different tissues.

The most strongly coexpressed gene was BAZ1A (Pearson's correlation coefficient .81), which is related to intellectual disabilities, mood disorders and some neoplasms in humans, according to www.disgenet.org (Li et al., 2016; Zaghlool et al., 2016).

We used ToppGene to identify GO terms relevant to genes that exhibited substantial coexpression with SRPK1 to understand the functional relevance of these genes throughout brain development. We mapped the top 3020 SRPK1‐linked genes to a coexpression network to evaluate the strength of the connections between the basis of both spatial and temporal coexpression trends with SRPK1 in the developing brain, and we may identify people. The phenotype associations early neurodevelopmental disorders of gait abnormalities including such as Charcot‐Marie‐Tooth disease (CMT) gait abnormalities, Congenital cataracts, facial dysmorphism, and neuropathy (CCFDN), Bryant‐Li‐Bhoj neurodevelopmental syndrome‐1 (BRYLIB1) neurodevelopmental delay, agenesis of corpus callosum related peripheral neuropathy, pulmonary atresia, global developmental disorder including (Basilicata‐Akhtar) Syndrome, and neurodegenerative disorder Amyotrophic lateral sclerosis (ALS) (Figure 3b). Most of the shared genes and their annotation reveal coexpression *p* value, *q* value Bonferroni, *q* value FDR B&Y is < .05 (Table S5; Figure 3b).

## 4 DISCUSSION

The significance of SRPK1 in the development and function of the human brain is now better understood owing to the thorough study of the temporally and spatially coexpressed correlates of SRPK1 expression. We present a thorough analysis of SRPK1 expression across anatomical areas and developmental stages in healthy human brain tissue. Analyzing SRPK1 expression in distinct brain areas showed elevated SRPK1 expression in the posteroventral (inferior) parietal cortex, the inferolateral temporal cortex, the primary motor cortex, the dorsal thalamus, and most of all, the ventrolateral prefrontal cortex during the early PCW. Later, expression gradually declined during adolescence and adulthood. However, no experimental data were found for SRPK1 expression in the developing human brain.

Immunohistochemistry revealed different neuron‐specific localization of SRPK1 in the human brain. According to previous studies, the human nervous system exhibits significant alternative splicing of the SRPK1 gene, resulting in various proteins with different neuronal functions. The areas with the most SRPK1 expression are the cerebral cortex (pyramidal neurons), cerebellar cortex (Purkinje cells), neostriatum (large polyhedral neurons), and entorhinal cortex (pyramidal neurons), with moderate to weak expression found in the hippocampus and hypothalamus (Mytilinaios et al., 2012). These prior findings are in agreement with our current findings of elevated expression in the temporal cortex and low expression in the hippocampus over the life span. It is now possible to measure the quantity of gene expression in individual cells from different brain regions owing to developments in single‐cell RNA sequencing (Tasic et al., 2016; Zeisel et al., 2015). Understanding how SRPK1 functions in various brain regions depending on the underlying cellular composition can considerably improve the tracking of SRPK1 expression in particular brain cells.

Empirical evidence shows a strong link between SRPK1, and genes primarily implicated in AD, amyotrophic lateral sclerosis, and Parkinson's disease, which may explain the high coexistence of neurodegenerative diseases. A previous study showed that SRPK1 controls the alternative splicing of exon 10, which plays a significant role in frontotemporal dementia and AD (Hartmann et al., 2001).

SRPK1 and the protein derived from one of the genes responsible for neurodevelopmental diseases are physically connected. Numerous studies have indicated that modifications in alternative splicing may play a role in the emergence of neurodevelopmental conditions, including autism spectrum disorders (ASD) and ID (Bustos et al., 2020; Shen et al., 2016; Stamova et al., 2013). A functional enhancement study of the significant coexpression of genes supports different functions for SRPK1 throughout development. The strong coexpression of SRPK1 with other genes within the network may be connected to elevated expression in early development. In contrast to other proteins, it is therefore uncertain that SRPK1‐specific protein interactions exist, but there may still be unidentified cell‐type‐specific interactions.

However, SRPK1 likely plays a vital role in deciding the response of particular cell types to stressful stimuli. Interestingly, during subchronic cell stress, SRPK1 was activated, likely resulting in the alternative splicing of microRNAs into antiapoptotic isoforms. The kinase is cleaved by caspases in response to prolonged fatal stress, ensuring that the apoptotic program is carried out faithfully in condemned cells (Kamachi et al., 2002).

These findings emphasize the significance of detailed maps of human brain expression for improved human‐to‐human translation and illustrate differences in SRPK1 expression between human brains. This study has two limitations that should be noted. First, the sample size of human donors was relatively modest, especially the number who provided information on life history factors (such as stressors) that can influence the expression of the SRPK1 receptor. However, as demonstrated by differential solid stability values, SRPK1 expression patterns differed among donors throughout life. Additionally, using the GTEx version 8 dataset for analysis, the present study revealed that genes significantly coexpressed with SRPK1 were also significantly coexpressed in neurodevelopmental disorders in adult humans. Second, while other techniques can quantify SRPK1 expression density directly, we utilized transcriptome information as a surrogate for gene expression levels (e.g., competitive binding receptor autoradiography). Using such methods, it is impossible to simultaneously evaluate the coexpression of more than a few receptors. Despite being a less direct method, transcriptome measurements make it easier to analyze the patterns of coexpression of genes for thousands of receptors and nonreceptor genes, which can assist in delineating the structures of the brain (M. Hawrylycz et al., 2015), whose SRPK1 expression was the subject of this paper.

## 5 CONCLUSION

Our findings suggest that SRPK1 is essential for the development and functioning of the human brain. These results support the need to assess SRPK1 expression and activity in human postmortem brain samples to understand the alternative splicing processes underlying neurodevelopmental disorders. This article presents evidence for distinctive SRPK1 expression patterns enriched in processes related to neurodevelopmental disorders across development. Additionally, it supports the need for clinical trials focusing on and monitoring the central nervous system. By analyzing the spatiotemporal gene expression profile of SRPK1 and identifying coexpressed genes, we found evidence that SRPK1 expression is involved in various neurodevelopmental and somatic events throughout the lifespan.

## AUTHOR CONTRIBUTIONS

Jing‐jing Wang and Sagor Kumar Roy designed the work. Sagor Kumar Roy extracted and analyzed the data. Yu‐ming Xu wrote this paper. Sagor Kumar Roy, Jing‐jing Wang, and Yu‐ming Xu interpreted the results and helped to revise the manuscript. All authors read and approved the final manuscript.

## CONFLICT OF INTEREST STATEMENT

No conflict of interest.

### PEER REVIEW

The peer review history for this article is available at https://publons.com/publon/10.1002/brb3.3341

## Supporting information

Table S1. RNA‐sequencing level with *Z* score of SRPK1 expression with its different structure and different age of brain (from http://www.brainspan.org/rnaseq/search/index.html).
Table S2. RNA‐sequencing level of SRPK1 expression with *Z* score of different structure of adult human postmortem brain (from https://human.brain‐map.org).
Table S3. Top 20 significant coexpression gene related to with SRPK1 (from https://genefriends.org).
Table S4. Top 28 coexpressed SRPK1 genes with different human organ expression (from https://fuma.ctglab.nl/).

## Data Availability

This article contains all data generated or analyzed during this investigation (and its Supplementary Information files). Here is following the dataset availability links: (1)  http://www.brainspan.org/rnaseq/search/index.html; RNA‐sequencing‐level with *Z* score of SRPK1 expression with its different structure and ages of the brain. (2) https://human.brain‐map.org; RNA‐sequencing‐level of SRPK1 expression with *Z* score of the adult human postmortem brain structures. (3) https://genefriends.org; top 20 significant coexpression genes related to SRPK1. (4) https://fuma.ctglab.nl/; top 28 coexpressed SRPK1 genes with different human organ expression 5. https://toppgene.cchmc.org/; using Toppgene to evaluate genes that exhibit substantial coexpression with SRPK1 for relevance in gene of phenotypes.
