# Supplementary material for: Spatiotemporal expression and coexpression patterns of SRPK1 in the human brain: A neurodevelopmental perspective
Source: Brain Behav. 2023 Dec 31;14(1):e3341. doi: 10.1002/brb3.3341 (PMC10757891; doi:10.1002/brb3.3341)
Supplement: Supplementary file 1 — Table S1. RNA‐sequencing level with Z score of SRPK1 expression with its different structure and different age of brain (from http://www.brainspan.org/rnaseq/search/index.html). Table S2. RNA‐sequencing level of SRPK1 expression with Z score of different structure of adult human postmortem brain (from https://human.brain‐map.org). Table S3. Top 20 significant coexpression gene related to with SRPK1 (from https://genefriends.org). Table S4. Top 28 coexpressed SRPK1 genes with different human organ expression (from https://fuma.ctglab.nl/). [file BRB3-14-e3341-s001.docx]

Supplementary Information

**Title: Spatiotemporal Expression and Co-expression of the SRPK1 in the Human Brain: A Neurodevelopmental Perspective**

Jingjing Wang#, Sagor Kumar Roy#, Yuming Xu*

*Corresponding author:

Dr. Yuming Xu, MD, PhD

Department of Neurology, The First Affiliated Hospital of Zhengzhou University, Zhengzhou University, Henan, Zhengzhou 450000, P. R. China

E-mail: [xuyuming@zzu.edu.cn](mailto:xuyuming@zzu.edu.cn)

Phone: +86-0371- 669113114

This file contains:

Supplementary Table 1

Supplementary Table 2

Supplementary Table 3

Supplementary Table 4

Supplementary Table 5

**Supplementary Table 1. Overview of the donor per age, expression with developmental stages following post conceptional week (PCW), child, adult according to different region of brain from Brainspan**

| **Donor ID** | **Age** | **Developmental stage** | **Brain location** | **Expression Z-score** |
| --- | --- | --- | --- | --- |
| H376.IIA.51 | 8 pcw | Post conceptional Week | amygdaloid complex | 3.9935 |
| H376.IIA.51 | 8 pcw | Post conceptional Week | caudal ganglionic eminence | 4.0723 |
| H376.IIA.51 | 8 pcw | Post conceptional Week | dorsolateral prefrontal cortex | 4.2489 |
| H376.IIA.51 | 8 pcw | Post conceptional Week | dorsal thalamus | 4.3635 |
| H376.IIA.51 | 8 pcw | Post conceptional Week | hippocampus (hippocampal formation) | 4.2118 |
| H376.IIA.51 | 8 pcw | Post conceptional Week | inferolateral temporal cortex (area TEv, area 20) | 3.9819 |
| H376.IIA.51 | 8 pcw | Post conceptional Week | lateral ganglionic eminence | 4.1104 |
| H376.IIA.51 | 8 pcw | Post conceptional Week | primary motor-sensory cortex (samples) | 4.2477 |
| H376.IIA.51 | 8 pcw | Post conceptional Week | anterior (rostral) cingulate (medial prefrontal) cortex | 4.345 |
| H376.IIA.51 | 8 pcw | Post conceptional Week | medial ganglionic eminence | 4.1516 |
| H376.IIA.51 | 8 pcw | Post conceptional Week | occipital neocortex | 4.2264 |
| H376.IIA.51 | 8 pcw | Post conceptional Week | orbital frontal cortex | 3.9079 |
| H376.IIA.51 | 8 pcw | Post conceptional Week | parietal neocortex | 4.0864 |
| H376.IIA.51 | 8 pcw | Post conceptional Week | posterior (caudal) superior temporal cortex (area 22c) | 4.1049 |
| H376.IIA.51 | 8 pcw | Post conceptional Week | upper (rostral) rhombic lip | 3.6359 |
| H376.IIA.51 | 8 pcw | Post conceptional Week | ventrolateral prefrontal cortex | 4.2341 |
| H376.IIA.50 | 9 pcw | Post conceptional Week | amygdaloid complex | 3.9674 |
| H376.IIA.50 | 9 pcw | Post conceptional Week | caudal ganglionic eminence | 4.0802 |
| H376.IIA.50 | 9 pcw | Post conceptional Week | dorsolateral prefrontal cortex | 4.0922 |
| H376.IIA.50 | 9 pcw | Post conceptional Week | dorsal thalamus | 4.4674 |
| H376.IIA.50 | 9 pcw | Post conceptional Week | hippocampus (hippocampal formation) | 4.1084 |
| H376.IIA.50 | 9 pcw | Post conceptional Week | lateral ganglionic eminence | 3.88 |
| H376.IIA.50 | 9 pcw | Post conceptional Week | primary motor-sensory cortex (samples) | 3.9641 |
| H376.IIA.50 | 9 pcw | Post conceptional Week | anterior (rostral) cingulate (medial prefrontal) cortex | 4.081 |
| H376.IIA.50 | 9 pcw | Post conceptional Week | medial ganglionic eminence | 4.0526 |
| H376.IIA.50 | 9 pcw | Post conceptional Week | occipital neocortex | 4.1664 |
| H376.IIA.50 | 9 pcw | Post conceptional Week | orbital frontal cortex | 4.0414 |
| H376.IIA.50 | 9 pcw | Post conceptional Week | parietal neocortex | 4.2181 |
| H376.IIA.50 | 9 pcw | Post conceptional Week | temporal neocortex | 4.2739 |
| H376.IIA.50 | 9 pcw | Post conceptional Week | upper (rostral) rhombic lip | 3.8568 |
| H376.IIB.50 | 12 pcw | Post conceptional Week | primary auditory cortex (core) | 4.1664 |
| H376.IIB.51 | 12 pcw | Post conceptional Week | primary auditory cortex (core) | 4.7479 |
| H376.IIB.52 | 12 pcw | Post conceptional Week | primary auditory cortex (core) | 4.5486 |
| H376.IIB.50 | 12 pcw | Post conceptional Week | amygdaloid complex | 3.767 |
| H376.IIB.51 | 12 pcw | Post conceptional Week | amygdaloid complex | 3.7976 |
| H376.IIB.52 | 12 pcw | Post conceptional Week | amygdaloid complex | 4.0887 |
| H376.IIB.51 | 12 pcw | Post conceptional Week | cerebellum | 4.2577 |
| H376.IIB.52 | 12 pcw | Post conceptional Week | cerebellar cortex | 4.3275 |
| H376.IIB.50 | 12 pcw | Post conceptional Week | dorsolateral prefrontal cortex | 4.2558 |
| H376.IIB.51 | 12 pcw | Post conceptional Week | dorsolateral prefrontal cortex | 4.7706 |
| H376.IIB.52 | 12 pcw | Post conceptional Week | dorsolateral prefrontal cortex | 4.6064 |
| H376.IIB.50 | 12 pcw | Post conceptional Week | dorsal thalamus | 3.6574 |
| H376.IIB.51 | 12 pcw | Post conceptional Week | dorsal thalamus | 4.4575 |
| H376.IIB.52 | 12 pcw | Post conceptional Week | dorsal thalamus | 4.5411 |
| H376.IIB.50 | 12 pcw | Post conceptional Week | hippocampus (hippocampal formation) | 3.7658 |
| H376.IIB.51 | 12 pcw | Post conceptional Week | hippocampus (hippocampal formation) | 4.0815 |
| H376.IIB.52 | 12 pcw | Post conceptional Week | hippocampus (hippocampal formation) | 4.2045 |
| H376.IIB.50 | 12 pcw | Post conceptional Week | posteroventral (inferior) parietal cortex | 4.3318 |
| H376.IIB.51 | 12 pcw | Post conceptional Week | posteroventral (inferior) parietal cortex | 4.6286 |
| H376.IIB.52 | 12 pcw | Post conceptional Week | posteroventral (inferior) parietal cortex | 4.8363 |
| H376.IIB.50 | 12 pcw | Post conceptional Week | inferolateral temporal cortex (area TEv, area 20) | 4.2258 |
| H376.IIB.51 | 12 pcw | Post conceptional Week | inferolateral temporal cortex (area TEv, area 20) | 4.5799 |
| H376.IIB.52 | 12 pcw | Post conceptional Week | inferolateral temporal cortex (area TEv, area 20) | 4.6497 |
| H376.IIB.50 | 12 pcw | Post conceptional Week | primary motor cortex (area M1, area 4) | 4.2658 |
| H376.IIB.51 | 12 pcw | Post conceptional Week | primary motor cortex (area M1, area 4) | 4.1409 |
| H376.IIB.52 | 12 pcw | Post conceptional Week | primary motor cortex (area M1, area 4) | 4.7226 |
| H376.IIB.50 | 12 pcw | Post conceptional Week | anterior (rostral) cingulate (medial prefrontal) cortex | 4.2387 |
| H376.IIB.52 | 12 pcw | Post conceptional Week | anterior (rostral) cingulate (medial prefrontal) cortex | 4.5025 |
| H376.IIB.50 | 12 pcw | Post conceptional Week | orbital frontal cortex | 4.2695 |
| H376.IIB.51 | 12 pcw | Post conceptional Week | orbital frontal cortex | 4.5918 |
| H376.IIB.52 | 12 pcw | Post conceptional Week | orbital frontal cortex | 4.5572 |
| H376.IIB.50 | 12 pcw | Post conceptional Week | primary somatosensory cortex (area S1, areas 3,1,2) | 4.3742 |
| H376.IIB.51 | 12 pcw | Post conceptional Week | primary somatosensory cortex (area S1, areas 3,1,2) | 4.6769 |
| H376.IIB.52 | 12 pcw | Post conceptional Week | primary somatosensory cortex (area S1, areas 3,1,2) | 4.6318 |
| H376.IIB.50 | 12 pcw | Post conceptional Week | posterior (caudal) superior temporal cortex (area 22c) | 4.294 |
| H376.IIB.51 | 12 pcw | Post conceptional Week | posterior (caudal) superior temporal cortex (area 22c) | 4.5166 |
| H376.IIB.50 | 12 pcw | Post conceptional Week | striatum | 4.5093 |
| H376.IIB.51 | 12 pcw | Post conceptional Week | striatum | 4.1765 |
| H376.IIB.52 | 12 pcw | Post conceptional Week | striatum | 3.8953 |
| H376.IIB.50 | 12 pcw | Post conceptional Week | primary visual cortex (striate cortex, area V1/17) | 4.5218 |
| H376.IIB.51 | 12 pcw | Post conceptional Week | primary visual cortex (striate cortex, area V1/17) | 4.2483 |
| H376.IIB.52 | 12 pcw | Post conceptional Week | primary visual cortex (striate cortex, area V1/17) | 4.7658 |
| H376.IIB.50 | 12 pcw | Post conceptional Week | ventrolateral prefrontal cortex | 4.3058 |
| H376.IIB.51 | 12 pcw | Post conceptional Week | ventrolateral prefrontal cortex | 4.5752 |
| H376.IIB.52 | 12 pcw | Post conceptional Week | ventrolateral prefrontal cortex | 4.7592 |
| H376.IIIA.50 | 13 pcw | Post conceptional Week | primary auditory cortex (core) | 4.165 |
| H376.IIIA.51 | 13 pcw | Post conceptional Week | primary auditory cortex (core) | 4.099 |
| H376.IIIA.52 | 13 pcw | Post conceptional Week | primary auditory cortex (core) | 4.7711 |
| H376.IIIA.50 | 13 pcw | Post conceptional Week | amygdaloid complex | 4.1891 |
| H376.IIIA.51 | 13 pcw | Post conceptional Week | amygdaloid complex | 3.7256 |
| H376.IIIA.52 | 13 pcw | Post conceptional Week | amygdaloid complex | 3.8913 |
| H376.IIIA.51 | 13 pcw | Post conceptional Week | cerebellum | 3.5763 |
| H376.IIIA.52 | 13 pcw | Post conceptional Week | cerebellum | 3.8355 |
| H376.IIIA.50 | 13 pcw | Post conceptional Week | dorsolateral prefrontal cortex | 4.5177 |
| H376.IIIA.51 | 13 pcw | Post conceptional Week | dorsolateral prefrontal cortex | 4.097 |
| H376.IIIA.52 | 13 pcw | Post conceptional Week | dorsolateral prefrontal cortex | 4.5857 |
| H376.IIIA.50 | 13 pcw | Post conceptional Week | hippocampus (hippocampal formation) | 3.8272 |
| H376.IIIA.51 | 13 pcw | Post conceptional Week | hippocampus (hippocampal formation) | 3.7493 |
| H376.IIIA.52 | 13 pcw | Post conceptional Week | hippocampus (hippocampal formation) | 3.9297 |
| H376.IIIA.50 | 13 pcw | Post conceptional Week | posteroventral (inferior) parietal cortex | 4.1842 |
| H376.IIIA.51 | 13 pcw | Post conceptional Week | posteroventral (inferior) parietal cortex | 4.3737 |
| H376.IIIA.52 | 13 pcw | Post conceptional Week | posteroventral (inferior) parietal cortex | 4.5065 |
| H376.IIIA.50 | 13 pcw | Post conceptional Week | inferolateral temporal cortex (area TEv, area 20) | 4.0273 |
| H376.IIIA.51 | 13 pcw | Post conceptional Week | inferolateral temporal cortex (area TEv, area 20) | 4.1993 |
| H376.IIIA.52 | 13 pcw | Post conceptional Week | inferolateral temporal cortex (area TEv, area 20) | 4.8802 |
| H376.IIIA.50 | 13 pcw | Post conceptional Week | primary motor cortex (area M1, area 4) | 4.4069 |
| H376.IIIA.51 | 13 pcw | Post conceptional Week | primary motor cortex (area M1, area 4) | 4.2046 |
| H376.IIIA.52 | 13 pcw | Post conceptional Week | primary motor cortex (area M1, area 4) | 4.4845 |
| H376.IIIA.51 | 13 pcw | Post conceptional Week | mediodorsal nucleus of thalamus | 4.0853 |
| H376.IIIA.50 | 13 pcw | Post conceptional Week | anterior (rostral) cingulate (medial prefrontal) cortex | 4.4797 |
| H376.IIIA.51 | 13 pcw | Post conceptional Week | anterior (rostral) cingulate (medial prefrontal) cortex | 4.0175 |
| H376.IIIA.52 | 13 pcw | Post conceptional Week | anterior (rostral) cingulate (medial prefrontal) cortex | 4.8588 |
| H376.IIIA.50 | 13 pcw | Post conceptional Week | orbital frontal cortex | 4.4796 |
| H376.IIIA.51 | 13 pcw | Post conceptional Week | orbital frontal cortex | 3.9325 |
| H376.IIIA.52 | 13 pcw | Post conceptional Week | orbital frontal cortex | 4.8524 |
| H376.IIIA.50 | 13 pcw | Post conceptional Week | primary somatosensory cortex (area S1, areas 3,1,2) | 4.2725 |
| H376.IIIA.51 | 13 pcw | Post conceptional Week | primary somatosensory cortex (area S1, areas 3,1,2) | 4.0681 |
| H376.IIIA.52 | 13 pcw | Post conceptional Week | primary somatosensory cortex (area S1, areas 3,1,2) | 4.6718 |
| H376.IIIA.50 | 13 pcw | Post conceptional Week | posterior (caudal) superior temporal cortex (area 22c) | 3.9753 |
| H376.IIIA.51 | 13 pcw | Post conceptional Week | posterior (caudal) superior temporal cortex (area 22c) | 4.2179 |
| H376.IIIA.50 | 13 pcw | Post conceptional Week | striatum | 3.6413 |
| H376.IIIA.51 | 13 pcw | Post conceptional Week | striatum | 3.4404 |
| H376.IIIA.52 | 13 pcw | Post conceptional Week | striatum | 4.1912 |
| H376.IIIA.50 | 13 pcw | Post conceptional Week | primary visual cortex (striate cortex, area V1/17) | 4.2863 |
| H376.IIIA.51 | 13 pcw | Post conceptional Week | primary visual cortex (striate cortex, area V1/17) | 4.1972 |
| H376.IIIA.52 | 13 pcw | Post conceptional Week | primary visual cortex (striate cortex, area V1/17) | 4.3833 |
| H376.IIIA.50 | 13 pcw | Post conceptional Week | ventrolateral prefrontal cortex | 4.3104 |
| H376.IIIA.51 | 13 pcw | Post conceptional Week | ventrolateral prefrontal cortex | 4.1245 |
| H376.IIIA.52 | 13 pcw | Post conceptional Week | ventrolateral prefrontal cortex | 4.9387 |
| H376.IIIB.50 | 16 pcw | Post conceptional Week | primary auditory cortex (core) | 3.5552 |
| H376.IIIB.51 | 16 pcw | Post conceptional Week | primary auditory cortex (core) | 3.9857 |
| H376.IIIB.52 | 16 pcw | Post conceptional Week | primary auditory cortex (core) | 4.1147 |
| H376.IIIB.51 | 16 pcw | Post conceptional Week | amygdaloid complex | 3.4027 |
| H376.IIIB.52 | 16 pcw | Post conceptional Week | amygdaloid complex | 3.8478 |
| H376.IIIB.51 | 16 pcw | Post conceptional Week | cerebellar cortex | 3.4693 |
| H376.IIIB.50 | 16 pcw | Post conceptional Week | dorsolateral prefrontal cortex | 3.7149 |
| H376.IIIB.51 | 16 pcw | Post conceptional Week | dorsolateral prefrontal cortex | 3.9905 |
| H376.IIIB.52 | 16 pcw | Post conceptional Week | dorsolateral prefrontal cortex | 4.807 |
| H376.IIIB.51 | 16 pcw | Post conceptional Week | hippocampus (hippocampal formation) | 3.5517 |
| H376.IIIB.52 | 16 pcw | Post conceptional Week | hippocampus (hippocampal formation) | 3.8336 |
| H376.IIIB.50 | 16 pcw | Post conceptional Week | posteroventral (inferior) parietal cortex | 3.839 |
| H376.IIIB.51 | 16 pcw | Post conceptional Week | posteroventral (inferior) parietal cortex | 4.0267 |
| H376.IIIB.52 | 16 pcw | Post conceptional Week | posteroventral (inferior) parietal cortex | 3.7479 |
| H376.IIIB.51 | 16 pcw | Post conceptional Week | inferolateral temporal cortex (area TEv, area 20) | 3.54 |
| H376.IIIB.52 | 16 pcw | Post conceptional Week | inferolateral temporal cortex (area TEv, area 20) | 3.9531 |
| H376.IIIB.51 | 16 pcw | Post conceptional Week | primary motor cortex (area M1, area 4) | 4.499 |
| H376.IIIB.52 | 16 pcw | Post conceptional Week | primary motor-sensory cortex (samples) | 4.2724 |
| H376.IIIB.50 | 16 pcw | Post conceptional Week | mediodorsal nucleus of thalamus | 3.984 |
| H376.IIIB.51 | 16 pcw | Post conceptional Week | mediodorsal nucleus of thalamus | 3.8451 |
| H376.IIIB.52 | 16 pcw | Post conceptional Week | mediodorsal nucleus of thalamus | 4.58 |
| H376.IIIB.50 | 16 pcw | Post conceptional Week | anterior (rostral) cingulate (medial prefrontal) cortex | 3.3399 |
| H376.IIIB.51 | 16 pcw | Post conceptional Week | anterior (rostral) cingulate (medial prefrontal) cortex | 3.8941 |
| H376.IIIB.52 | 16 pcw | Post conceptional Week | anterior (rostral) cingulate (medial prefrontal) cortex | 4.2767 |
| H376.IIIB.50 | 16 pcw | Post conceptional Week | orbital frontal cortex | 3.7342 |
| H376.IIIB.51 | 16 pcw | Post conceptional Week | orbital frontal cortex | 4.1047 |
| H376.IIIB.51 | 16 pcw | Post conceptional Week | primary somatosensory cortex (area S1, areas 3,1,2) | 4.2262 |
| H376.IIIB.50 | 16 pcw | Post conceptional Week | posterior (caudal) superior temporal cortex (area 22c) | 3.466 |
| H376.IIIB.51 | 16 pcw | Post conceptional Week | posterior (caudal) superior temporal cortex (area 22c) | 3.5593 |
| H376.IIIB.52 | 16 pcw | Post conceptional Week | posterior (caudal) superior temporal cortex (area 22c) | 3.8759 |
| H376.IIIB.50 | 16 pcw | Post conceptional Week | striatum | 3.3669 |
| H376.IIIB.51 | 16 pcw | Post conceptional Week | striatum | 3.6435 |
| H376.IIIB.52 | 16 pcw | Post conceptional Week | striatum | 3.8532 |
| H376.IIIB.50 | 16 pcw | Post conceptional Week | primary visual cortex (striate cortex, area V1/17) | 3.6335 |
| H376.IIIB.51 | 16 pcw | Post conceptional Week | primary visual cortex (striate cortex, area V1/17) | 3.9254 |
| H376.IIIB.52 | 16 pcw | Post conceptional Week | primary visual cortex (striate cortex, area V1/17) | 4.1611 |
| H376.IIIB.50 | 16 pcw | Post conceptional Week | ventrolateral prefrontal cortex | 3.7924 |
| H376.IIIB.51 | 16 pcw | Post conceptional Week | ventrolateral prefrontal cortex | 4.0843 |
| H376.IIIB.52 | 16 pcw | Post conceptional Week | ventrolateral prefrontal cortex | 4.2324 |
| H376.IIIB.53 | 17 pcw | Post conceptional Week | primary auditory cortex (core) | 3.9963 |
| H376.IIIB.53 | 17 pcw | Post conceptional Week | amygdaloid complex | 3.6526 |
| H376.IIIB.53 | 17 pcw | Post conceptional Week | cerebellar cortex | 3.7493 |
| H376.IIIB.53 | 17 pcw | Post conceptional Week | dorsolateral prefrontal cortex | 3.8541 |
| H376.IIIB.53 | 17 pcw | Post conceptional Week | hippocampus (hippocampal formation) | 2.8794 |
| H376.IIIB.53 | 17 pcw | Post conceptional Week | posteroventral (inferior) parietal cortex | 4.2241 |
| H376.IIIB.53 | 17 pcw | Post conceptional Week | primary motor-sensory cortex (samples) | 3.9943 |
| H376.IIIB.53 | 17 pcw | Post conceptional Week | mediodorsal nucleus of thalamus | 3.7966 |
| H376.IIIB.53 | 17 pcw | Post conceptional Week | anterior (rostral) cingulate (medial prefrontal) cortex | 4.0254 |
| H376.IIIB.53 | 17 pcw | Post conceptional Week | orbital frontal cortex | 4.0487 |
| H376.IIIB.53 | 17 pcw | Post conceptional Week | posterior (caudal) superior temporal cortex (area 22c) | 3.3945 |
| H376.IIIB.53 | 17 pcw | Post conceptional Week | striatum | 3.0506 |
| H376.IIIB.53 | 17 pcw | Post conceptional Week | primary visual cortex (striate cortex, area V1/17) | 3.5764 |
| H376.IIIB.53 | 17 pcw | Post conceptional Week | ventrolateral prefrontal cortex | 3.6105 |
| H376.IV.53 | 19 pcw | Post conceptional Week | primary auditory cortex (core) | 3.2926 |
| H376.IV.53 | 19 pcw | Post conceptional Week | dorsolateral prefrontal cortex | 3.7276 |
| H376.IV.53 | 19 pcw | Post conceptional Week | hippocampus (hippocampal formation) | 2.7088 |
| H376.IV.53 | 19 pcw | Post conceptional Week | posteroventral (inferior) parietal cortex | 3.1612 |
| H376.IV.53 | 19 pcw | Post conceptional Week | primary motor-sensory cortex (samples) | 3.4009 |
| H376.IV.53 | 19 pcw | Post conceptional Week | mediodorsal nucleus of thalamus | 3.4377 |
| H376.IV.53 | 19 pcw | Post conceptional Week | anterior (rostral) cingulate (medial prefrontal) cortex | 3.5651 |
| H376.IV.53 | 19 pcw | Post conceptional Week | posterior (caudal) superior temporal cortex (area 22c) | 3.1909 |
| H376.IV.53 | 19 pcw | Post conceptional Week | striatum | 3.0456 |
| H376.IV.53 | 19 pcw | Post conceptional Week | primary visual cortex (striate cortex, area V1/17) | 3.0071 |
| H376.IV.53 | 19 pcw | Post conceptional Week | ventrolateral prefrontal cortex | 3.7015 |
| H376.IV.54 | 21 pcw | Post conceptional Week | amygdaloid complex | 3.8397 |
| H376.IV.51 | 21 pcw | Post conceptional Week | cerebellar cortex | 3.6286 |
| H376.IV.54 | 21 pcw | Post conceptional Week | cerebellar cortex | 3.7188 |
| H376.IV.54 | 21 pcw | Post conceptional Week | dorsolateral prefrontal cortex | 3.635 |
| H376.IV.54 | 21 pcw | Post conceptional Week | hippocampus (hippocampal formation) | 3.2819 |
| H376.IV.54 | 21 pcw | Post conceptional Week | posteroventral (inferior) parietal cortex | 4.155 |
| H376.IV.51 | 21 pcw | Post conceptional Week | inferolateral temporal cortex (area TEv, area 20) | 3.142 |
| H376.IV.54 | 21 pcw | Post conceptional Week | inferolateral temporal cortex (area TEv, area 20) | 4.2205 |
| H376.IV.54 | 21 pcw | Post conceptional Week | primary motor cortex (area M1, area 4) | 4.1166 |
| H376.IV.54 | 21 pcw | Post conceptional Week | anterior (rostral) cingulate (medial prefrontal) cortex | 3.9527 |
| H376.IV.54 | 21 pcw | Post conceptional Week | orbital frontal cortex | 4.0849 |
| H376.IV.54 | 21 pcw | Post conceptional Week | primary somatosensory cortex (area S1, areas 3,1,2) | 4.3226 |
| H376.IV.54 | 21 pcw | Post conceptional Week | posterior (caudal) superior temporal cortex (area 22c) | 3.7989 |
| H376.IV.54 | 21 pcw | Post conceptional Week | striatum | 3.2091 |
| H376.IV.54 | 21 pcw | Post conceptional Week | primary visual cortex (striate cortex, area V1/17) | 3.8398 |
| H376.IV.54 | 21 pcw | Post conceptional Week | ventrolateral prefrontal cortex | 3.4976 |
| H376.IV.50 | 24 pcw | Post conceptional Week | primary auditory cortex (core) | 4.2565 |
| H376.IV.50 | 24 pcw | Post conceptional Week | amygdaloid complex | 3.5539 |
| H376.IV.50 | 24 pcw | Post conceptional Week | cerebellar cortex | 3.9629 |
| H376.IV.50 | 24 pcw | Post conceptional Week | dorsolateral prefrontal cortex | 4.5042 |
| H376.IV.50 | 24 pcw | Post conceptional Week | hippocampus (hippocampal formation) | 3.7147 |
| H376.IV.50 | 24 pcw | Post conceptional Week | posteroventral (inferior) parietal cortex | 4.1919 |
| H376.IV.50 | 24 pcw | Post conceptional Week | inferolateral temporal cortex (area TEv, area 20) | 4.3128 |
| H376.IV.50 | 24 pcw | Post conceptional Week | primary motor cortex (area M1, area 4) | 4.0906 |
| H376.IV.50 | 24 pcw | Post conceptional Week | mediodorsal nucleus of thalamus | 3.8831 |
| H376.IV.50 | 24 pcw | Post conceptional Week | anterior (rostral) cingulate (medial prefrontal) cortex | 4.2616 |
| H376.IV.50 | 24 pcw | Post conceptional Week | orbital frontal cortex | 4.4594 |
| H376.IV.50 | 24 pcw | Post conceptional Week | primary somatosensory cortex (area S1, areas 3,1,2) | 4.1196 |
| H376.IV.50 | 24 pcw | Post conceptional Week | posterior (caudal) superior temporal cortex (area 22c) | 4.0312 |
| H376.IV.50 | 24 pcw | Post conceptional Week | striatum | 3.7316 |
| H376.IV.50 | 24 pcw | Post conceptional Week | primary visual cortex (striate cortex, area V1/17) | 4.051 |
| H376.IV.50 | 24 pcw | Post conceptional Week | ventrolateral prefrontal cortex | 4.3503 |
| H376.V.51 | 25 pcw | Post conceptional Week | primary auditory cortex (core) | 1.951 |
| H376.V.52 | 26 pcw | Post conceptional Week | dorsolateral prefrontal cortex | 1.5394 |
| H376.V.52 | 26 pcw | Post conceptional Week | posterior (caudal) superior temporal cortex (area 22c) | 4.2045 |
| H376.V.52 | 26 pcw | Post conceptional Week | primary visual cortex (striate cortex, area V1/17) | 1.787 |
| H376.V.50 | 35 pcw | Post conceptional Week | cerebellar cortex | 3.2195 |
| H376.V.50 | 35 pcw | Post conceptional Week | ventrolateral prefrontal cortex | 3.2638 |
| H376.V.53 | 37 pcw | Post conceptional Week | primary auditory cortex (core) | 3.212 |
| H376.V.53 | 37 pcw | Post conceptional Week | amygdaloid complex | 1.9581 |
| H376.V.53 | 37 pcw | Post conceptional Week | cerebellar cortex | 2.9379 |
| H376.V.53 | 37 pcw | Post conceptional Week | dorsolateral prefrontal cortex | 3.2124 |
| H376.V.53 | 37 pcw | Post conceptional Week | hippocampus (hippocampal formation) | 1.7434 |
| H376.V.53 | 37 pcw | Post conceptional Week | posteroventral (inferior) parietal cortex | 2.9719 |
| H376.V.53 | 37 pcw | Post conceptional Week | inferolateral temporal cortex (area TEv, area 20) | 2.9825 |
| H376.V.53 | 37 pcw | Post conceptional Week | primary motor cortex (area M1, area 4) | 2.3988 |
| H376.V.53 | 37 pcw | Post conceptional Week | mediodorsal nucleus of thalamus | 1.8848 |
| H376.V.53 | 37 pcw | Post conceptional Week | anterior (rostral) cingulate (medial prefrontal) cortex | 2.681 |
| H376.V.53 | 37 pcw | Post conceptional Week | orbital frontal cortex | 2.904 |
| H376.V.53 | 37 pcw | Post conceptional Week | primary somatosensory cortex (area S1, areas 3,1,2) | 3.016 |
| H376.V.53 | 37 pcw | Post conceptional Week | posterior (caudal) superior temporal cortex (area 22c) | 3.1924 |
| H376.V.53 | 37 pcw | Post conceptional Week | striatum | 2.0058 |
| H376.V.53 | 37 pcw | Post conceptional Week | primary visual cortex (striate cortex, area V1/17) | 3.627 |
| H376.V.53 | 37 pcw | Post conceptional Week | ventrolateral prefrontal cortex | 2.9708 |
| H376.VI.51 | 4 mos | Child | primary auditory cortex (core) | 2.0365 |
| H376.VI.52 | 4 mos | Child | primary auditory cortex (core) | 3.5832 |
| H376.VI.50 | 4 mos | Child | amygdaloid complex | 2.3179 |
| H376.VI.51 | 4 mos | Child | amygdaloid complex | 2.0614 |
| H376.VI.52 | 4 mos | Child | amygdaloid complex | 2.6017 |
| H376.VI.50 | 4 mos | Child | cerebellar cortex | 3.5209 |
| H376.VI.52 | 4 mos | Child | cerebellar cortex | 3.2561 |
| H376.VI.51 | 4 mos | Child | dorsolateral prefrontal cortex | 2.1023 |
| H376.VI.52 | 4 mos | Child | dorsolateral prefrontal cortex | 3.4449 |
| H376.VI.50 | 4 mos | Child | hippocampus (hippocampal formation) | 2.4465 |
| H376.VI.52 | 4 mos | Child | hippocampus (hippocampal formation) | 2.0854 |
| H376.VI.52 | 4 mos | Child | posteroventral (inferior) parietal cortex | 3.5535 |
| H376.VI.50 | 4 mos | Child | inferolateral temporal cortex (area TEv, area 20) | 3.6073 |
| H376.VI.51 | 4 mos | Child | inferolateral temporal cortex (area TEv, area 20) | 2.4009 |
| H376.VI.52 | 4 mos | Child | inferolateral temporal cortex (area TEv, area 20) | 3.2155 |
| H376.VI.51 | 4 mos | Child | primary motor cortex (area M1, area 4) | 2.141 |
| H376.VI.52 | 4 mos | Child | primary motor cortex (area M1, area 4) | 3.2306 |
| H376.VI.50 | 4 mos | Child | mediodorsal nucleus of thalamus | 3.1966 |
| H376.VI.52 | 4 mos | Child | mediodorsal nucleus of thalamus | 2.9985 |
| H376.VI.50 | 4 mos | Child | anterior (rostral) cingulate (medial prefrontal) cortex | 3.2963 |
| H376.VI.52 | 4 mos | Child | anterior (rostral) cingulate (medial prefrontal) cortex | 3.3074 |
| H376.VI.51 | 4 mos | Child | orbital frontal cortex | 1.9772 |
| H376.VI.52 | 4 mos | Child | orbital frontal cortex | 3.3346 |
| H376.VI.52 | 4 mos | Child | primary somatosensory cortex (area S1, areas 3,1,2) | 3.3876 |
| H376.VI.50 | 4 mos | Child | posterior (caudal) superior temporal cortex (area 22c) | 3.5324 |
| H376.VI.51 | 4 mos | Child | posterior (caudal) superior temporal cortex (area 22c) | 2.2206 |
| H376.VI.52 | 4 mos | Child | posterior (caudal) superior temporal cortex (area 22c) | 3.3639 |
| H376.VI.50 | 4 mos | Child | striatum | 2.6411 |
| H376.VI.52 | 4 mos | Child | striatum | 2.423 |
| H376.VI.50 | 4 mos | Child | primary visual cortex (striate cortex, area V1/17) | 3.4067 |
| H376.VI.52 | 4 mos | Child | primary visual cortex (striate cortex, area V1/17) | 3.9993 |
| H376.VI.51 | 4 mos | Child | ventrolateral prefrontal cortex | 2.2705 |
| H376.VI.52 | 4 mos | Child | ventrolateral prefrontal cortex | 3.2198 |
| H376.VII.51 | 10 mos | Child | cerebellar cortex | 3.869 |
| H376.VII.51 | 10 mos | Child | dorsolateral prefrontal cortex | 3.7432 |
| H376.VII.51 | 10 mos | Child | posteroventral (inferior) parietal cortex | 3.7327 |
| H376.VII.51 | 10 mos | Child | inferolateral temporal cortex (area TEv, area 20) | 3.3857 |
| H376.VII.51 | 10 mos | Child | mediodorsal nucleus of thalamus | 2.885 |
| H376.VII.51 | 10 mos | Child | anterior (rostral) cingulate (medial prefrontal) cortex | 3.326 |
| H376.VII.51 | 10 mos | Child | orbital frontal cortex | 3.634 |
| H376.VII.51 | 10 mos | Child | primary somatosensory cortex (area S1, areas 3,1,2) | 3.9711 |
| H376.VII.51 | 10 mos | Child | posterior (caudal) superior temporal cortex (area 22c) | 3.6453 |
| H376.VII.51 | 10 mos | Child | primary visual cortex (striate cortex, area V1/17) | 3.9915 |
| H376.VIII.51 | 1 yrs | Child | primary auditory cortex (core) | 3.6604 |
| H376.VIII.51 | 1 yrs | Child | amygdaloid complex | 2.7471 |
| H376.VIII.51 | 1 yrs | Child | cerebellar cortex | 3.9368 |
| H376.VIII.51 | 1 yrs | Child | dorsolateral prefrontal cortex | 3.4665 |
| H376.VIII.51 | 1 yrs | Child | hippocampus (hippocampal formation) | 2.938 |
| H376.VIII.51 | 1 yrs | Child | posteroventral (inferior) parietal cortex | 3.7501 |
| H376.VIII.51 | 1 yrs | Child | inferolateral temporal cortex (area TEv, area 20) | 3.5765 |
| H376.VIII.51 | 1 yrs | Child | primary motor cortex (area M1, area 4) | 3.2644 |
| H376.VIII.51 | 1 yrs | Child | mediodorsal nucleus of thalamus | 2.9099 |
| H376.VIII.51 | 1 yrs | Child | anterior (rostral) cingulate (medial prefrontal) cortex | 2.9765 |
| H376.VIII.51 | 1 yrs | Child | orbital frontal cortex | 3.1802 |
| H376.VIII.51 | 1 yrs | Child | primary somatosensory cortex (area S1, areas 3,1,2) | 3.6175 |
| H376.VIII.51 | 1 yrs | Child | posterior (caudal) superior temporal cortex (area 22c) | 3.5489 |
| H376.VIII.51 | 1 yrs | Child | striatum | 3.0602 |
| H376.VIII.51 | 1 yrs | Child | primary visual cortex (striate cortex, area V1/17) | 4.1175 |
| H376.VIII.51 | 1 yrs | Child | ventrolateral prefrontal cortex | 3.558 |
| H376.VIII.53 | 2 yrs | Child | cerebellar cortex | 2.2528 |
| H376.VIII.53 | 2 yrs | Child | dorsolateral prefrontal cortex | 1.3412 |
| H376.VIII.53 | 2 yrs | Child | hippocampus (hippocampal formation) | 1.29 |
| H376.VIII.53 | 2 yrs | Child | posteroventral (inferior) parietal cortex | 1.1101 |
| H376.VIII.53 | 2 yrs | Child | inferolateral temporal cortex (area TEv, area 20) | 0.9669 |
| H376.VIII.53 | 2 yrs | Child | mediodorsal nucleus of thalamus | 1.2927 |
| H376.VIII.53 | 2 yrs | Child | anterior (rostral) cingulate (medial prefrontal) cortex | 0.9192 |
| H376.VIII.53 | 2 yrs | Child | orbital frontal cortex | 1.0323 |
| H376.VIII.53 | 2 yrs | Child | primary somatosensory cortex (area S1, areas 3,1,2) | 1.2142 |
| H376.VIII.53 | 2 yrs | Child | posterior (caudal) superior temporal cortex (area 22c) | 1.4451 |
| H376.VIII.53 | 2 yrs | Child | primary visual cortex (striate cortex, area V1/17) | 1.398 |
| H376.VIII.53 | 2 yrs | Child | ventrolateral prefrontal cortex | 1.3941 |
| H376.VIII.52 | 3 yrs | Child | primary auditory cortex (core) | 2.2333 |
| H376.VIII.54 | 3 yrs | Child | primary auditory cortex (core) | 1.7899 |
| H376.VIII.52 | 3 yrs | Child | amygdaloid complex | 1.6355 |
| H376.VIII.54 | 3 yrs | Child | amygdaloid complex | 1.5394 |
| H376.VIII.52 | 3 yrs | Child | cerebellar cortex | 2.9199 |
| H376.VIII.54 | 3 yrs | Child | cerebellar cortex | 3.4213 |
| H376.VIII.54 | 3 yrs | Child | dorsolateral prefrontal cortex | 1.6274 |
| H376.VIII.54 | 3 yrs | Child | hippocampus (hippocampal formation) | 1.6746 |
| H376.VIII.52 | 3 yrs | Child | posteroventral (inferior) parietal cortex | 2.4387 |
| H376.VIII.54 | 3 yrs | Child | posteroventral (inferior) parietal cortex | 1.8437 |
| H376.VIII.52 | 3 yrs | Child | inferolateral temporal cortex (area TEv, area 20) | 2.0703 |
| H376.VIII.54 | 3 yrs | Child | inferolateral temporal cortex (area TEv, area 20) | 1.951 |
| H376.VIII.52 | 3 yrs | Child | primary motor cortex (area M1, area 4) | 2.2363 |
| H376.VIII.54 | 3 yrs | Child | primary motor cortex (area M1, area 4) | 1.7733 |
| H376.VIII.52 | 3 yrs | Child | mediodorsal nucleus of thalamus | 1.6445 |
| H376.VIII.54 | 3 yrs | Child | anterior (rostral) cingulate (medial prefrontal) cortex | 1.8386 |
| H376.VIII.54 | 3 yrs | Child | orbital frontal cortex | 1.9662 |
| H376.VIII.54 | 3 yrs | Child | primary somatosensory cortex (area S1, areas 3,1,2) | 1.7354 |
| H376.VIII.52 | 3 yrs | Child | posterior (caudal) superior temporal cortex (area 22c) | 2.1522 |
| H376.VIII.54 | 3 yrs | Child | posterior (caudal) superior temporal cortex (area 22c) | 1.9541 |
| H376.VIII.52 | 3 yrs | Child | striatum | 1.503 |
| H376.VIII.52 | 3 yrs | Child | primary visual cortex (striate cortex, area V1/17) | 2.4153 |
| H376.VIII.54 | 3 yrs | Child | primary visual cortex (striate cortex, area V1/17) | 1.787 |
| H376.VIII.52 | 3 yrs | Child | ventrolateral prefrontal cortex | 2.3046 |
| H376.VIII.54 | 3 yrs | Child | ventrolateral prefrontal cortex | 1.7176 |
| H376.VIII.50 | 4 yrs | Child | amygdaloid complex | 2.169 |
| H376.VIII.50 | 4 yrs | Child | cerebellar cortex | 3.3902 |
| H376.VIII.50 | 4 yrs | Child | dorsolateral prefrontal cortex | 2.1031 |
| H376.VIII.50 | 4 yrs | Child | mediodorsal nucleus of thalamus | 2.5658 |
| H376.VIII.50 | 4 yrs | Child | posterior (caudal) superior temporal cortex (area 22c) | 2.8446 |
| H376.VIII.50 | 4 yrs | Child | striatum | 2.6697 |
| H376.VIII.50 | 4 yrs | Child | ventrolateral prefrontal cortex | 2.5417 |
| H376.IX.51 | 8 yrs | Child | primary auditory cortex (core) | 3.0552 |
| H376.IX.52 | 8 yrs | Child | primary auditory cortex (core) | 2.7067 |
| H376.IX.51 | 8 yrs | Child | amygdaloid complex | 2.3981 |
| H376.IX.52 | 8 yrs | Child | amygdaloid complex | 2.2225 |
| H376.IX.51 | 8 yrs | Child | cerebellar cortex | 2.9263 |
| H376.IX.52 | 8 yrs | Child | cerebellar cortex | 3.1673 |
| H376.IX.51 | 8 yrs | Child | dorsolateral prefrontal cortex | 2.5744 |
| H376.IX.52 | 8 yrs | Child | dorsolateral prefrontal cortex | 2.3324 |
| H376.IX.51 | 8 yrs | Child | hippocampus (hippocampal formation) | 2.6055 |
| H376.IX.52 | 8 yrs | Child | hippocampus (hippocampal formation) | 2.5841 |
| H376.IX.51 | 8 yrs | Child | posteroventral (inferior) parietal cortex | 2.8084 |
| H376.IX.52 | 8 yrs | Child | posteroventral (inferior) parietal cortex | 2.7857 |
| H376.IX.51 | 8 yrs | Child | inferolateral temporal cortex (area TEv, area 20) | 2.7344 |
| H376.IX.52 | 8 yrs | Child | inferolateral temporal cortex (area TEv, area 20) | 2.8266 |
| H376.IX.51 | 8 yrs | Child | primary motor cortex (area M1, area 4) | 2.7795 |
| H376.IX.51 | 8 yrs | Child | mediodorsal nucleus of thalamus | 2.1204 |
| H376.IX.51 | 8 yrs | Child | anterior (rostral) cingulate (medial prefrontal) cortex | 2.3319 |
| H376.IX.52 | 8 yrs | Child | anterior (rostral) cingulate (medial prefrontal) cortex | 2.3922 |
| H376.IX.51 | 8 yrs | Child | orbital frontal cortex | 2.7268 |
| H376.IX.51 | 8 yrs | Child | primary somatosensory cortex (area S1, areas 3,1,2) | 3.0535 |
| H376.IX.51 | 8 yrs | Child | posterior (caudal) superior temporal cortex (area 22c) | 2.896 |
| H376.IX.52 | 8 yrs | Child | posterior (caudal) superior temporal cortex (area 22c) | 2.8128 |
| H376.IX.51 | 8 yrs | Child | striatum | 2.4961 |
| H376.IX.51 | 8 yrs | Child | primary visual cortex (striate cortex, area V1/17) | 3.297 |
| H376.IX.52 | 8 yrs | Child | primary visual cortex (striate cortex, area V1/17) | 3.0649 |
| H376.IX.51 | 8 yrs | Child | ventrolateral prefrontal cortex | 2.6267 |
| H376.IX.52 | 8 yrs | Child | ventrolateral prefrontal cortex | 2.8573 |
| H376.IX.50 | 11 yrs | Child | primary auditory cortex (core) | 3.4141 |
| H376.IX.50 | 11 yrs | Child | amygdaloid complex | 2.3254 |
| H376.IX.50 | 11 yrs | Child | cerebellar cortex | 3.4287 |
| H376.IX.50 | 11 yrs | Child | dorsolateral prefrontal cortex | 2.8973 |
| H376.IX.50 | 11 yrs | Child | hippocampus (hippocampal formation) | 2.7159 |
| H376.IX.50 | 11 yrs | Child | posteroventral (inferior) parietal cortex | 3.2558 |
| H376.IX.50 | 11 yrs | Child | inferolateral temporal cortex (area TEv, area 20) | 3.1346 |
| H376.IX.50 | 11 yrs | Child | primary motor cortex (area M1, area 4) | 2.9348 |
| H376.IX.50 | 11 yrs | Child | anterior (rostral) cingulate (medial prefrontal) cortex | 2.7998 |
| H376.IX.50 | 11 yrs | Child | orbital frontal cortex | 2.9275 |
| H376.IX.50 | 11 yrs | Child | primary somatosensory cortex (area S1, areas 3,1,2) | 3.1361 |
| H376.IX.50 | 11 yrs | Child | posterior (caudal) superior temporal cortex (area 22c) | 3.0997 |
| H376.IX.50 | 11 yrs | Child | primary visual cortex (striate cortex, area V1/17) | 3.2675 |
| H376.IX.50 | 11 yrs | Child | ventrolateral prefrontal cortex | 2.9751 |
| H376.X.51 | 13 yrs | Child | primary auditory cortex (core) | 2.819 |
| H376.X.51 | 13 yrs | Child | amygdaloid complex | 2.0591 |
| H376.X.51 | 13 yrs | Child | cerebellar cortex | 3.4627 |
| H376.X.51 | 13 yrs | Child | dorsolateral prefrontal cortex | 2.6866 |
| H376.X.51 | 13 yrs | Child | hippocampus (hippocampal formation) | 1.7132 |
| H376.X.51 | 13 yrs | Child | posteroventral (inferior) parietal cortex | 2.4719 |
| H376.X.51 | 13 yrs | Child | inferolateral temporal cortex (area TEv, area 20) | 2.4919 |
| H376.X.51 | 13 yrs | Child | primary motor cortex (area M1, area 4) | 2.8159 |
| H376.X.51 | 13 yrs | Child | mediodorsal nucleus of thalamus | 1.6066 |
| H376.X.51 | 13 yrs | Child | anterior (rostral) cingulate (medial prefrontal) cortex | 2.3959 |
| H376.X.51 | 13 yrs | Child | orbital frontal cortex | 2.4853 |
| H376.X.51 | 13 yrs | Child | primary somatosensory cortex (area S1, areas 3,1,2) | 2.8013 |
| H376.X.51 | 13 yrs | Child | posterior (caudal) superior temporal cortex (area 22c) | 2.5809 |
| H376.X.51 | 13 yrs | Child | striatum | 2.1846 |
| H376.X.51 | 13 yrs | Child | primary visual cortex (striate cortex, area V1/17) | 2.6428 |
| H376.X.51 | 13 yrs | Child | ventrolateral prefrontal cortex | 2.8347 |
| H376.X.50 | 15 yrs | Child | amygdaloid complex | 2.7721 |
| H376.X.50 | 15 yrs | Child | cerebellar cortex | 3.655 |
| H376.X.50 | 15 yrs | Child | posteroventral (inferior) parietal cortex | 3.376 |
| H376.X.50 | 15 yrs | Child | inferolateral temporal cortex (area TEv, area 20) | 3.2848 |
| H376.X.50 | 15 yrs | Child | posterior (caudal) superior temporal cortex (area 22c) | 3.512 |
| H376.X.53 | 18 yrs | Adult | primary auditory cortex (core) | 1.7188 |
| H376.X.53 | 18 yrs | Adult | cerebellar cortex | 2.5304 |
| H376.X.53 | 18 yrs | Adult | dorsolateral prefrontal cortex | 2.4442 |
| H376.X.53 | 18 yrs | Adult | hippocampus (hippocampal formation) | 1.4912 |
| H376.X.53 | 18 yrs | Adult | posteroventral (inferior) parietal cortex | 1.7112 |
| H376.X.53 | 18 yrs | Adult | inferolateral temporal cortex (area TEv, area 20) | 1.8965 |
| H376.X.53 | 18 yrs | Adult | primary motor cortex (area M1, area 4) | 1.7118 |
| H376.X.53 | 18 yrs | Adult | anterior (rostral) cingulate (medial prefrontal) cortex | 1.3518 |
| H376.X.53 | 18 yrs | Adult | orbital frontal cortex | 1.9138 |
| H376.X.53 | 18 yrs | Adult | primary somatosensory cortex (area S1, areas 3,1,2) | 1.5827 |
| H376.X.53 | 18 yrs | Adult | posterior (caudal) superior temporal cortex (area 22c) | 1.5663 |
| H376.X.53 | 18 yrs | Adult | primary visual cortex (striate cortex, area V1/17) | 1.7292 |
| H376.X.53 | 18 yrs | Adult | ventrolateral prefrontal cortex | 1.9275 |
| H376.X.52 | 19 yrs | Adult | primary auditory cortex (core) | 3.5388 |
| H376.X.52 | 19 yrs | Adult | amygdaloid complex | 1.9256 |
| H376.X.52 | 19 yrs | Adult | cerebellar cortex | 3.2348 |
| H376.X.52 | 19 yrs | Adult | dorsolateral prefrontal cortex | 2.83 |
| H376.X.52 | 19 yrs | Adult | hippocampus (hippocampal formation) | 2.2311 |
| H376.X.52 | 19 yrs | Adult | posteroventral (inferior) parietal cortex | 3.3617 |
| H376.X.52 | 19 yrs | Adult | inferolateral temporal cortex (area TEv, area 20) | 2.5199 |
| H376.X.52 | 19 yrs | Adult | primary motor cortex (area M1, area 4) | 2.8247 |
| H376.X.52 | 19 yrs | Adult | mediodorsal nucleus of thalamus | 2.3633 |
| H376.X.52 | 19 yrs | Adult | anterior (rostral) cingulate (medial prefrontal) cortex | 2.8042 |
| H376.X.52 | 19 yrs | Adult | orbital frontal cortex | 2.9559 |
| H376.X.52 | 19 yrs | Adult | primary somatosensory cortex (area S1, areas 3,1,2) | 3.2508 |
| H376.X.52 | 19 yrs | Adult | posterior (caudal) superior temporal cortex (area 22c) | 3.093 |
| H376.X.52 | 19 yrs | Adult | striatum | 2.7877 |
| H376.X.52 | 19 yrs | Adult | primary visual cortex (striate cortex, area V1/17) | 3.301 |
| H376.X.52 | 19 yrs | Adult | ventrolateral prefrontal cortex | 2.8132 |
| H376.XI.60 | 21 yrs | Adult | primary auditory cortex (core) | 3.5036 |
| H376.XI.60 | 21 yrs | Adult | amygdaloid complex | 3.0098 |
| H376.XI.60 | 21 yrs | Adult | cerebellar cortex | 3.7822 |
| H376.XI.60 | 21 yrs | Adult | dorsolateral prefrontal cortex | 3.1428 |
| H376.XI.60 | 21 yrs | Adult | hippocampus (hippocampal formation) | 2.5703 |
| H376.XI.60 | 21 yrs | Adult | posteroventral (inferior) parietal cortex | 3.3242 |
| H376.XI.60 | 21 yrs | Adult | inferolateral temporal cortex (area TEv, area 20) | 3.2364 |
| H376.XI.60 | 21 yrs | Adult | primary motor cortex (area M1, area 4) | 3.2477 |
| H376.XI.60 | 21 yrs | Adult | mediodorsal nucleus of thalamus | 3.0537 |
| H376.XI.60 | 21 yrs | Adult | anterior (rostral) cingulate (medial prefrontal) cortex | 2.7893 |
| H376.XI.60 | 21 yrs | Adult | orbital frontal cortex | 3.2373 |
| H376.XI.60 | 21 yrs | Adult | primary somatosensory cortex (area S1, areas 3,1,2) | 3.6166 |
| H376.XI.60 | 21 yrs | Adult | posterior (caudal) superior temporal cortex (area 22c) | 3.181 |
| H376.XI.60 | 21 yrs | Adult | striatum | 2.534 |
| H376.XI.60 | 21 yrs | Adult | primary visual cortex (striate cortex, area V1/17) | 3.703 |
| H376.XI.60 | 21 yrs | Adult | ventrolateral prefrontal cortex | 3.2891 |
| H376.XI.50 | 23 yrs | Adult | primary auditory cortex (core) | 2.643 |
| H376.XI.50 | 23 yrs | Adult | amygdaloid complex | 1.9777 |
| H376.XI.50 | 23 yrs | Adult | cerebellar cortex | 3.2469 |
| H376.XI.50 | 23 yrs | Adult | hippocampus (hippocampal formation) | 2.1339 |
| H376.XI.50 | 23 yrs | Adult | posteroventral (inferior) parietal cortex | 2.9731 |
| H376.XI.50 | 23 yrs | Adult | inferolateral temporal cortex (area TEv, area 20) | 2.3191 |
| H376.XI.50 | 23 yrs | Adult | primary motor cortex (area M1, area 4) | 2.5538 |
| H376.XI.50 | 23 yrs | Adult | mediodorsal nucleus of thalamus | 1.7463 |
| H376.XI.50 | 23 yrs | Adult | anterior (rostral) cingulate (medial prefrontal) cortex | 2.7355 |
| H376.XI.50 | 23 yrs | Adult | orbital frontal cortex | 2.4815 |
| H376.XI.50 | 23 yrs | Adult | primary somatosensory cortex (area S1, areas 3,1,2) | 2.7152 |
| H376.XI.50 | 23 yrs | Adult | posterior (caudal) superior temporal cortex (area 22c) | 2.581 |
| H376.XI.50 | 23 yrs | Adult | striatum | 2.004 |
| H376.XI.50 | 23 yrs | Adult | ventrolateral prefrontal cortex | 2.6462 |
| H376.XI.52 | 30 yrs | Adult | primary auditory cortex (core) | 2.1953 |
| H376.XI.52 | 30 yrs | Adult | amygdaloid complex | 2.2372 |
| H376.XI.52 | 30 yrs | Adult | cerebellar cortex | 3.2028 |
| H376.XI.52 | 30 yrs | Adult | dorsolateral prefrontal cortex | 2.8069 |
| H376.XI.52 | 30 yrs | Adult | hippocampus (hippocampal formation) | 2.1872 |
| H376.XI.52 | 30 yrs | Adult | posteroventral (inferior) parietal cortex | 2.5039 |
| H376.XI.52 | 30 yrs | Adult | inferolateral temporal cortex (area TEv, area 20) | 2.4472 |
| H376.XI.52 | 30 yrs | Adult | primary motor cortex (area M1, area 4) | 2.5074 |
| H376.XI.52 | 30 yrs | Adult | mediodorsal nucleus of thalamus | 2.241 |
| H376.XI.52 | 30 yrs | Adult | anterior (rostral) cingulate (medial prefrontal) cortex | 2.1736 |
| H376.XI.52 | 30 yrs | Adult | orbital frontal cortex | 2.228 |
| H376.XI.52 | 30 yrs | Adult | primary somatosensory cortex (area S1, areas 3,1,2) | 2.7191 |
| H376.XI.52 | 30 yrs | Adult | posterior (caudal) superior temporal cortex (area 22c) | 2.8243 |
| H376.XI.52 | 30 yrs | Adult | striatum | 1.9947 |
| H376.XI.52 | 30 yrs | Adult | primary visual cortex (striate cortex, area V1/17) | 2.9493 |
| H376.XI.52 | 30 yrs | Adult | ventrolateral prefrontal cortex | 2.299 |
| H376.XI.53 | 36 yrs | Adult | primary auditory cortex (core) | 2.9639 |
| H376.XI.53 | 36 yrs | Adult | amygdaloid complex | 2.1346 |
| H376.XI.53 | 36 yrs | Adult | cerebellar cortex | 3.3626 |
| H376.XI.53 | 36 yrs | Adult | dorsolateral prefrontal cortex | 2.956 |
| H376.XI.53 | 36 yrs | Adult | hippocampus (hippocampal formation) | 2.425 |
| H376.XI.53 | 36 yrs | Adult | posteroventral (inferior) parietal cortex | 2.7274 |
| H376.XI.53 | 36 yrs | Adult | inferolateral temporal cortex (area TEv, area 20) | 2.7016 |
| H376.XI.53 | 36 yrs | Adult | primary motor cortex (area M1, area 4) | 2.6366 |
| H376.XI.53 | 36 yrs | Adult | mediodorsal nucleus of thalamus | 2.429 |
| H376.XI.53 | 36 yrs | Adult | anterior (rostral) cingulate (medial prefrontal) cortex | 2.5798 |
| H376.XI.53 | 36 yrs | Adult | orbital frontal cortex | 2.7799 |
| H376.XI.53 | 36 yrs | Adult | primary somatosensory cortex (area S1, areas 3,1,2) | 2.8406 |
| H376.XI.53 | 36 yrs | Adult | posterior (caudal) superior temporal cortex (area 22c) | 3.0484 |
| H376.XI.53 | 36 yrs | Adult | striatum | 2.5075 |
| H376.XI.53 | 36 yrs | Adult | primary visual cortex (striate cortex, area V1/17) | 3.2943 |
| H376.XI.53 | 36 yrs | Adult | ventrolateral prefrontal cortex | 2.8961 |
| H376.XI.54 | 37 yrs | Adult | primary auditory cortex (core) | 3.4032 |
| H376.XI.54 | 37 yrs | Adult | amygdaloid complex | 2.8016 |
| H376.XI.54 | 37 yrs | Adult | cerebellar cortex | 3.3483 |
| H376.XI.54 | 37 yrs | Adult | dorsolateral prefrontal cortex | 3.2373 |
| H376.XI.54 | 37 yrs | Adult | hippocampus (hippocampal formation) | 2.9594 |
| H376.XI.54 | 37 yrs | Adult | posteroventral (inferior) parietal cortex | 3.1205 |
| H376.XI.54 | 37 yrs | Adult | inferolateral temporal cortex (area TEv, area 20) | 3.2989 |
| H376.XI.54 | 37 yrs | Adult | primary motor cortex (area M1, area 4) | 3.135 |
| H376.XI.54 | 37 yrs | Adult | mediodorsal nucleus of thalamus | 2.5705 |
| H376.XI.54 | 37 yrs | Adult | anterior (rostral) cingulate (medial prefrontal) cortex | 3.4812 |
| H376.XI.54 | 37 yrs | Adult | orbital frontal cortex | 2.8383 |
| H376.XI.54 | 37 yrs | Adult | primary somatosensory cortex (area S1, areas 3,1,2) | 3.2786 |
| H376.XI.54 | 37 yrs | Adult | posterior (caudal) superior temporal cortex (area 22c) | 2.9667 |
| H376.XI.54 | 37 yrs | Adult | striatum | 2.679 |
| H376.XI.54 | 37 yrs | Adult | primary visual cortex (striate cortex, area V1/17) | 3.3069 |
| H376.XI.54 | 37 yrs | Adult | ventrolateral prefrontal cortex | 3.1535 |
| H376.XI.56 | 40 yrs | Adult | primary auditory cortex (core) | 2.6175 |
| H376.XI.56 | 40 yrs | Adult | amygdaloid complex | 2.1959 |
| H376.XI.56 | 40 yrs | Adult | cerebellar cortex | 2.9674 |
| H376.XI.56 | 40 yrs | Adult | dorsolateral prefrontal cortex | 2.7405 |
| H376.XI.56 | 40 yrs | Adult | hippocampus (hippocampal formation) | 2.3705 |
| H376.XI.56 | 40 yrs | Adult | posteroventral (inferior) parietal cortex | 3.3383 |
| H376.XI.56 | 40 yrs | Adult | inferolateral temporal cortex (area TEv, area 20) | 2.6292 |
| H376.XI.56 | 40 yrs | Adult | primary motor cortex (area M1, area 4) | 2.6486 |
| H376.XI.56 | 40 yrs | Adult | mediodorsal nucleus of thalamus | 1.879 |
| H376.XI.56 | 40 yrs | Adult | orbital frontal cortex | 3.0972 |
| H376.XI.56 | 40 yrs | Adult | primary somatosensory cortex (area S1, areas 3,1,2) | 2.8826 |
| H376.XI.56 | 40 yrs | Adult | posterior (caudal) superior temporal cortex (area 22c) | 3.3467 |
| H376.XI.56 | 40 yrs | Adult | striatum | 2.2052 |
| H376.XI.56 | 40 yrs | Adult | primary visual cortex (striate cortex, area V1/17) | 3.2811 |
| H376.XI.56 | 40 yrs | Adult | ventrolateral prefrontal cortex | 3.278 |

**Supplementary Table 2: Summery of the donor ID, expression with according to different region of brain from AHBA**

| **Donor ID** | **Location of brain** | **Expression Z score** |
| --- | --- | --- |
| 309335438 | hippocampus (hippocampal formation) | -0.5994 |
| 309335439 | hippocampus (hippocampal formation) | -0.6393 |
| 309335440 | hippocampus (hippocampal formation) | -0.583 |
| 309335441 | hippocampus (hippocampal formation) | -0.5847 |
| 309335442 | hippocampus (hippocampal formation) | -0.4735 |
| 309335443 | hippocampus (hippocampal formation) | -1.3335 |
| 309335444 | hippocampus (hippocampal formation) | -1.4933 |
| 309335445 | hippocampus (hippocampal formation) | -0.7007 |
| 309335446 | hippocampus (hippocampal formation) | -0.7411 |
| 309335447 | hippocampus (hippocampal formation) | -1.0684 |
| 309335449 | hippocampus (hippocampal formation) | -1.3603 |
| 309335451 | hippocampus (hippocampal formation) | -0.9502 |
| 309335452 | hippocampus (hippocampal formation) | -1.0299 |
| 309335454 | hippocampus (hippocampal formation) | -0.7214 |
| 309335455 | hippocampus (hippocampal formation) | 0.1883 |
| 309335456 | hippocampus (hippocampal formation) | -0.4366 |
| 309335457 | hippocampus (hippocampal formation) | -0.5756 |
| 309335460 | hippocampus (hippocampal formation) | -0.9155 |
| 309335461 | hippocampus (hippocampal formation) | -1.8214 |
| 309335462 | hippocampus (hippocampal formation) | -1.1524 |
| 309335463 | hippocampus (hippocampal formation) | -0.2808 |
| 309335464 | hippocampus (hippocampal formation) | -0.7279 |
| 309335465 | hippocampus (hippocampal formation) | -1.3809 |
| 309335466 | hippocampus (hippocampal formation) | -0.1427 |
| 309335467 | hippocampus (hippocampal formation) | -0.7083 |
| 309335468 | hippocampus (hippocampal formation) | -0.5805 |
| 309335469 | hippocampus (hippocampal formation) | -0.9026 |
| 309335470 | hippocampus (hippocampal formation) | -1.3829 |
| 309335471 | hippocampus (hippocampal formation) | -0.4872 |
| 309335474 | hippocampus (hippocampal formation) | -0.4978 |
| 309335475 | hippocampus (hippocampal formation) | -0.7423 |
| 309335478 | hippocampus (hippocampal formation) | -1.2795 |
| 309335479 | hippocampus (hippocampal formation) | -0.5052 |
| 309335481 | hippocampus (hippocampal formation) | -0.9807 |
| 309335482 | hippocampus (hippocampal formation) | -0.6697 |
| 309335483 | hippocampus (hippocampal formation) | -1.219 |
| 309335484 | hippocampus (hippocampal formation) | -1.0079 |
| 309335485 | hippocampus (hippocampal formation) | -0.5703 |
| 309335486 | hippocampus (hippocampal formation) | -0.678 |
| 309335487 | hippocampus (hippocampal formation) | -1.459 |
| 309335488 | hippocampus (hippocampal formation) | -0.4096 |
| 309335489 | hippocampus (hippocampal formation) | -0.2407 |
| 309335490 | hippocampus (hippocampal formation) | -0.752 |
| 309335491 | hippocampus (hippocampal formation) | -0.7291 |
| 309335492 | hippocampus (hippocampal formation) | -0.9606 |
| 309335493 | hippocampus (hippocampal formation) | -0.454 |
| 309335494 | hippocampus (hippocampal formation) | -1.1652 |
| 309335495 | hippocampus (hippocampal formation) | -0.1931 |
| 309335496 | hippocampus (hippocampal formation) | -0.717 |
| 309335497 | hippocampus (hippocampal formation) | -0.9646 |
| 326765648 | hippocampus (hippocampal formation) | -1.1117 |
| 326765649 | hippocampus (hippocampal formation) | -0.9079 |
| 326765650 | hippocampus (hippocampal formation) | -1.27 |
| 326765651 | hippocampus (hippocampal formation) | -1.0833 |
| 326765652 | hippocampus (hippocampal formation) | -0.9986 |
| 326765653 | hippocampus (hippocampal formation) | -0.8098 |
| 326765654 | hippocampus (hippocampal formation) | -0.6202 |
| 326765655 | hippocampus (hippocampal formation) | -0.9242 |
| 326765656 | hippocampus (hippocampal formation) | -0.6014 |
| 326765657 | hippocampus (hippocampal formation) | -0.4364 |
| 326765658 | hippocampus (hippocampal formation) | -0.8239 |
| 326765659 | hippocampus (hippocampal formation) | -0.3785 |
| 326765661 | hippocampus (hippocampal formation) | -0.3587 |
| 326765662 | hippocampus (hippocampal formation) | -1.2017 |
| 326765663 | hippocampus (hippocampal formation) | -0.9319 |
| 326765665 | hippocampus (hippocampal formation) | -0.2735 |
| 326765667 | hippocampus (hippocampal formation) | -0.4727 |
| 326765668 | hippocampus (hippocampal formation) | -0.967 |
| 326765669 | hippocampus (hippocampal formation) | -1.4383 |
| 326765670 | hippocampus (hippocampal formation) | -0.6532 |
| 326765671 | hippocampus (hippocampal formation) | -0.8183 |
| 326765672 | hippocampus (hippocampal formation) | -0.7457 |
| 326765673 | hippocampus (hippocampal formation) | -1.4383 |
| 326765674 | hippocampus (hippocampal formation) | -1.2351 |
| 326765675 | hippocampus (hippocampal formation) | 0.0274 |
| 326765676 | hippocampus (hippocampal formation) | -0.6227 |
| 326765677 | hippocampus (hippocampal formation) | -0.4416 |
| 326765678 | hippocampus (hippocampal formation) | -0.6103 |
| 326765679 | hippocampus (hippocampal formation) | -1.5719 |
| 326765680 | hippocampus (hippocampal formation) | -0.1289 |
| 326765681 | hippocampus (hippocampal formation) | -1.1176 |
| 326765682 | hippocampus (hippocampal formation) | -1.3252 |
| 326765683 | hippocampus (hippocampal formation) | -0.7311 |
| 326765686 | hippocampus (hippocampal formation) | -0.3731 |
| 326765687 | hippocampus (hippocampal formation) | -1.3124 |
| 326765688 | hippocampus (hippocampal formation) | -0.6966 |
| 326765689 | hippocampus (hippocampal formation) | -0.7575 |
| 467056391 | hippocampus (hippocampal formation) | -1.8255 |
| 467056397 | hippocampus (hippocampal formation) | -0.3034 |
| 467056405 | hippocampus (hippocampal formation) | -0.1682 |
| 467056406 | hippocampus (hippocampal formation) | -0.9736 |
| 467056407 | hippocampus (hippocampal formation) | -1.5698 |
| 467056408 | hippocampus (hippocampal formation) | -0.6552 |
| 467056409 | hippocampus (hippocampal formation) | -0.3076 |
| 309335438 | parietal neocortex | 0.864 |
| 309335439 | parietal neocortex | 0.8225 |
| 309335441 | parietal neocortex | 0.488 |
| 309335443 | parietal neocortex | 0.8916 |
| 309335444 | parietal neocortex | 0.2044 |
| 309335446 | parietal neocortex | 1.5549 |
| 309335447 | parietal neocortex | 0.8798 |
| 309335450 | parietal neocortex | -0.6655 |
| 309335451 | parietal neocortex | 0.2959 |
| 309335452 | parietal neocortex | 0.7423 |
| 309335453 | parietal neocortex | -0.6737 |
| 309335454 | parietal neocortex | 0.2856 |
| 309335455 | parietal neocortex | 2.0791 |
| 309335456 | parietal neocortex | 0.3837 |
| 309335457 | parietal neocortex | 1.6355 |
| 309335458 | parietal neocortex | 0.7697 |
| 309335459 | parietal neocortex | 0.3041 |
| 309335460 | parietal neocortex | 0.1723 |
| 309335461 | parietal neocortex | 0.9289 |
| 309335463 | parietal neocortex | 1.3321 |
| 309335465 | parietal neocortex | 0.7154 |
| 309335467 | parietal neocortex | -0.7335 |
| 309335468 | parietal neocortex | 0.803 |
| 309335469 | parietal neocortex | 0.316 |
| 309335470 | parietal neocortex | 0.2324 |
| 309335471 | parietal neocortex | 0.8966 |
| 309335474 | parietal neocortex | 0.6553 |
| 309335475 | parietal neocortex | 0.5745 |
| 309335476 | parietal neocortex | 1.3103 |
| 309335477 | parietal neocortex | 0.0548 |
| 309335479 | parietal neocortex | 0.7046 |
| 309335480 | parietal neocortex | 0.9673 |
| 309335482 | parietal neocortex | 0.9683 |
| 309335483 | parietal neocortex | 0.4479 |
| 309335484 | parietal neocortex | 0.657 |
| 309335485 | parietal neocortex | 0.3188 |
| 309335487 | parietal neocortex | 0.2337 |
| 309335488 | parietal neocortex | 1.6674 |
| 309335489 | parietal neocortex | 0.0788 |
| 309335490 | parietal neocortex | 1.1311 |
| 309335491 | parietal neocortex | -0.0057 |
| 309335492 | parietal neocortex | 0.0843 |
| 309335493 | parietal neocortex | 0.9207 |
| 309335494 | parietal neocortex | 0.6987 |
| 309335495 | parietal neocortex | 1.3061 |
| 309335496 | parietal neocortex | 0.8575 |
| 309335497 | parietal neocortex | 0.862 |
| 326765648 | parietal neocortex | 0.2558 |
| 326765649 | parietal neocortex | 0.2922 |
| 326765650 | parietal neocortex | 1.0361 |
| 326765651 | parietal neocortex | 0.1502 |
| 326765653 | parietal neocortex | 0.4514 |
| 326765654 | parietal neocortex | 0.8544 |
| 326765655 | parietal neocortex | 0.5398 |
| 326765656 | parietal neocortex | 0.0068 |
| 326765657 | parietal neocortex | 1.2516 |
| 326765658 | parietal neocortex | 0.8306 |
| 326765659 | parietal neocortex | 0.6866 |
| 326765660 | parietal neocortex | 1.1181 |
| 326765661 | parietal neocortex | 0.9459 |
| 326765662 | parietal neocortex | 0.9485 |
| 326765663 | parietal neocortex | 0.5962 |
| 326765664 | parietal neocortex | 1.1754 |
| 326765666 | parietal neocortex | 0.8437 |
| 326765667 | parietal neocortex | 0.2122 |
| 326765668 | parietal neocortex | -0.1873 |
| 326765669 | parietal neocortex | 0.0832 |
| 326765670 | parietal neocortex | 1.0776 |
| 326765673 | parietal neocortex | 0.7359 |
| 326765674 | parietal neocortex | 0.1525 |
| 326765675 | parietal neocortex | 0.5311 |
| 326765676 | parietal neocortex | 0.202 |
| 326765677 | parietal neocortex | 0.7662 |
| 326765678 | parietal neocortex | 0.1644 |
| 326765679 | parietal neocortex | 1.4765 |
| 326765680 | parietal neocortex | 1.1433 |
| 326765681 | parietal neocortex | 0.0447 |
| 326765682 | parietal neocortex | 0.4184 |
| 326765683 | parietal neocortex | 0.8978 |
| 326765684 | parietal neocortex | 0.4718 |
| 326765685 | parietal neocortex | 0.9798 |
| 326765686 | parietal neocortex | 0.647 |
| 326765687 | parietal neocortex | -0.4672 |
| 326765688 | parietal neocortex | 1.2913 |
| 326765689 | parietal neocortex | 0.8973 |
| 467056391 | parietal neocortex | 0.1161 |
| 467056397 | parietal neocortex | 0.5449 |
| 467056406 | parietal neocortex | 0.1584 |
| 467056407 | parietal neocortex | 0.3669 |
| 467056408 | parietal neocortex | 0.5092 |
| 467056409 | parietal neocortex | 1.9707 |
| 309335438 | temporal neocortex | 1.2648 |
| 309335439 | temporal neocortex | 1.5465 |
| 309335440 | temporal neocortex | 0.6688 |
| 309335441 | temporal neocortex | 0.8155 |
| 309335443 | temporal neocortex | 0.9276 |
| 309335444 | temporal neocortex | 0.3666 |
| 309335445 | temporal neocortex | 0.5281 |
| 309335446 | temporal neocortex | 1.5736 |
| 309335447 | temporal neocortex | 1.1584 |
| 309335448 | temporal neocortex | -0.1059 |
| 309335449 | temporal neocortex | 0.247 |
| 309335450 | temporal neocortex | 0.5235 |
| 309335451 | temporal neocortex | 0.5577 |
| 309335452 | temporal neocortex | 0.7056 |
| 309335453 | temporal neocortex | -0.0371 |
| 309335454 | temporal neocortex | 2.2751 |
| 309335455 | temporal neocortex | 1.3754 |
| 309335456 | temporal neocortex | 0.7379 |
| 309335457 | temporal neocortex | 2.1771 |
| 309335458 | temporal neocortex | 1.3402 |
| 309335459 | temporal neocortex | 1.0273 |
| 309335460 | temporal neocortex | 0.6636 |
| 309335461 | temporal neocortex | 0.1136 |
| 309335462 | temporal neocortex | 0.2509 |
| 309335463 | temporal neocortex | 0.783 |
| 309335465 | temporal neocortex | 0.8211 |
| 309335466 | temporal neocortex | 0.9921 |
| 309335467 | temporal neocortex | 0.5101 |
| 309335468 | temporal neocortex | 1.0357 |
| 309335469 | temporal neocortex | 0.7507 |
| 309335470 | temporal neocortex | 0.6854 |
| 309335471 | temporal neocortex | 1.4012 |
| 309335474 | temporal neocortex | 0.4684 |
| 309335475 | temporal neocortex | 1.0018 |
| 309335476 | temporal neocortex | 1.2895 |
| 309335477 | temporal neocortex | 0.2154 |
| 309335480 | temporal neocortex | 0.7174 |
| 309335481 | temporal neocortex | 1.2017 |
| 309335482 | temporal neocortex | 1.2058 |
| 309335483 | temporal neocortex | 1.1626 |
| 309335484 | temporal neocortex | 0.9445 |
| 309335485 | temporal neocortex | 0.1321 |
| 309335486 | temporal neocortex | 1.4636 |
| 309335487 | temporal neocortex | -0.0009 |
| 309335488 | temporal neocortex | 1.6315 |
| 309335489 | temporal neocortex | 0.2622 |
| 309335490 | temporal neocortex | 1.2963 |
| 309335491 | temporal neocortex | 1.2205 |
| 309335492 | temporal neocortex | 0.392 |
| 309335493 | temporal neocortex | 0.8365 |
| 309335494 | temporal neocortex | 0.991 |
| 309335495 | temporal neocortex | 0.8955 |
| 309335496 | temporal neocortex | 0.8007 |
| 309335497 | temporal neocortex | 0.8278 |
| 326765648 | temporal neocortex | 0.5917 |
| 326765649 | temporal neocortex | 0.9905 |
| 326765650 | temporal neocortex | 1.0745 |
| 326765651 | temporal neocortex | -0.0049 |
| 326765653 | temporal neocortex | 0.9446 |
| 326765654 | temporal neocortex | 0.9504 |
| 326765655 | temporal neocortex | 0.7688 |
| 326765656 | temporal neocortex | 0.5811 |
| 326765657 | temporal neocortex | 1.5135 |
| 326765658 | temporal neocortex | 1.165 |
| 326765659 | temporal neocortex | 0.868 |
| 326765660 | temporal neocortex | 0.9693 |
| 326765661 | temporal neocortex | 1.1348 |
| 326765662 | temporal neocortex | -0.1164 |
| 326765663 | temporal neocortex | 1.25 |
| 326765665 | temporal neocortex | 1.6895 |
| 326765666 | temporal neocortex | 1.181 |
| 326765667 | temporal neocortex | 0.5017 |
| 326765668 | temporal neocortex | 0.7115 |
| 326765669 | temporal neocortex | 0.4301 |
| 326765670 | temporal neocortex | 0.858 |
| 326765672 | temporal neocortex | -0.4442 |
| 326765673 | temporal neocortex | 0.5525 |
| 326765674 | temporal neocortex | 0.2216 |
| 326765675 | temporal neocortex | 1.5734 |
| 326765676 | temporal neocortex | 0.8697 |
| 326765678 | temporal neocortex | 1.5991 |
| 326765679 | temporal neocortex | 1.479 |
| 326765680 | temporal neocortex | 1.2921 |
| 326765681 | temporal neocortex | -0.1219 |
| 326765682 | temporal neocortex | 0.3469 |
| 326765683 | temporal neocortex | 1.1644 |
| 326765684 | temporal neocortex | 0.5913 |
| 326765685 | temporal neocortex | 1.7168 |
| 326765686 | temporal neocortex | 1.1412 |
| 326765687 | temporal neocortex | 0.1472 |
| 326765688 | temporal neocortex | 0.5874 |
| 326765689 | temporal neocortex | 1.6264 |
| 467056391 | temporal neocortex | 0.5884 |
| 467056397 | temporal neocortex | 0.6058 |
| 467056405 | temporal neocortex | 1.3656 |
| 467056406 | temporal neocortex | 0.6662 |
| 467056407 | temporal neocortex | 0.2267 |
| 467056408 | temporal neocortex | 1.4315 |
| 467056409 | temporal neocortex | 1.2052 |
| 309335438 | white matter of forebrain | -2.783 |
| 309335439 | white matter of forebrain | 0.664 |
| 309335441 | white matter of forebrain | -2.4825 |
| 309335443 | white matter of forebrain | 0.2101 |
| 309335444 | white matter of forebrain | -0.0127 |
| 309335445 | white matter of forebrain | -0.391 |
| 309335446 | white matter of forebrain | 0.897 |
| 309335447 | white matter of forebrain | -0.7239 |
| 309335450 | white matter of forebrain | -1.9465 |
| 309335451 | white matter of forebrain | -1.2929 |
| 309335452 | white matter of forebrain | -1.4615 |
| 309335453 | white matter of forebrain | -1.6368 |
| 309335454 | white matter of forebrain | 0.7208 |
| 309335455 | white matter of forebrain | -0.3495 |
| 309335456 | white matter of forebrain | 0.0448 |
| 309335457 | white matter of forebrain | 0.5498 |
| 309335458 | white matter of forebrain | -1.0762 |
| 309335459 | white matter of forebrain | -0.1203 |
| 309335460 | white matter of forebrain | -2.4686 |
| 309335461 | white matter of forebrain | -1.0126 |
| 309335463 | white matter of forebrain | 0.9408 |
| 309335465 | white matter of forebrain | -2.5291 |
| 309335467 | white matter of forebrain | -1.1281 |
| 309335468 | white matter of forebrain | -1.0036 |
| 309335469 | white matter of forebrain | -0.3871 |
| 309335470 | white matter of forebrain | -0.8263 |
| 309335471 | white matter of forebrain | -0.3009 |
| 309335474 | white matter of forebrain | -0.8827 |
| 309335475 | white matter of forebrain | -0.5859 |
| 309335476 | white matter of forebrain | -1.2219 |
| 309335477 | white matter of forebrain | -0.1023 |
| 309335479 | white matter of forebrain | -0.1371 |
| 309335480 | white matter of forebrain | -0.2178 |
| 309335482 | white matter of forebrain | -0.9814 |
| 309335483 | white matter of forebrain | 0.544 |
| 309335484 | white matter of forebrain | -0.4674 |
| 309335485 | white matter of forebrain | -0.8791 |
| 309335486 | white matter of forebrain | 0.1889 |
| 309335487 | white matter of forebrain | -2.4743 |
| 309335488 | white matter of forebrain | -0.9258 |
| 309335489 | white matter of forebrain | -0.9556 |
| 309335490 | white matter of forebrain | -0.3718 |
| 309335491 | white matter of forebrain | -0.2542 |
| 309335492 | white matter of forebrain | -0.5432 |
| 309335493 | white matter of forebrain | -0.4495 |
| 309335494 | white matter of forebrain | -0.4014 |
| 309335495 | white matter of forebrain | 0.6529 |
| 309335496 | white matter of forebrain | -1.5272 |
| 309335497 | white matter of forebrain | -2.3917 |
| 326765648 | white matter of forebrain | -1.4877 |
| 326765649 | white matter of forebrain | -0.0144 |
| 326765650 | white matter of forebrain | 1.0093 |
| 326765651 | white matter of forebrain | -0.7913 |
| 326765653 | white matter of forebrain | -1.7182 |
| 326765654 | white matter of forebrain | -1.3239 |
| 326765655 | white matter of forebrain | 0.771 |
| 326765656 | white matter of forebrain | -2.2709 |
| 326765657 | white matter of forebrain | -0.0811 |
| 326765658 | white matter of forebrain | -0.2777 |
| 326765659 | white matter of forebrain | -2.1029 |
| 326765660 | white matter of forebrain | 1.0062 |
| 326765661 | white matter of forebrain | 0.752 |
| 326765662 | white matter of forebrain | -2.1087 |
| 326765663 | white matter of forebrain | -1.6346 |
| 326765664 | white matter of forebrain | -0.3127 |
| 326765666 | white matter of forebrain | -0.8772 |
| 326765667 | white matter of forebrain | -0.7108 |
| 326765668 | white matter of forebrain | -1.7283 |
| 326765669 | white matter of forebrain | -0.9106 |
| 326765670 | white matter of forebrain | 0.2812 |
| 326765673 | white matter of forebrain | -0.1682 |
| 326765674 | white matter of forebrain | -0.371 |
| 326765675 | white matter of forebrain | 0.1609 |
| 326765676 | white matter of forebrain | -1.8414 |
| 326765677 | white matter of forebrain | -1.5008 |
| 326765678 | white matter of forebrain | -1.9243 |
| 326765679 | white matter of forebrain | -0.1088 |
| 326765680 | white matter of forebrain | -0.1454 |
| 326765681 | white matter of forebrain | -1.8106 |
| 326765682 | white matter of forebrain | -2.4272 |
| 326765683 | white matter of forebrain | -1.3062 |
| 326765684 | white matter of forebrain | -1.2333 |
| 326765685 | white matter of forebrain | 0.5291 |
| 326765686 | white matter of forebrain | -0.8944 |
| 326765687 | white matter of forebrain | -1.7389 |
| 326765688 | white matter of forebrain | 0.5415 |
| 326765689 | white matter of forebrain | 1.0088 |
| 467056391 | white matter of forebrain | -0.7179 |
| 467056397 | white matter of forebrain | -0.0549 |
| 467056406 | white matter of forebrain | -1.319 |
| 467056407 | white matter of forebrain | -1.2955 |
| 467056408 | white matter of forebrain | -2.3566 |
| 467056409 | white matter of forebrain | 1.2129 |

**Supplementary Table 3. GTEx 8 version expressed of different tissues SRPK1 co-expression patterns top 30 gene data-set gathered from** [**https://genefriends.org**](https://genefriends.org)

| **Co-expressed gene** | **Pearson Correlation** | **Gene Biotype** |
| --- | --- | --- |
| BAZ1A | 0.813656539 | Protein coding |
| CYB5R4 | 0.793188102 | Protein coding |
| PPP4C | 0.790180026 | Protein coding |
| CAPZA1 | 0.779806199 | Protein coding |
| TRIM25 | 0.77756467 | Protein coding |
| USP15 | 0.774728306 | Protein coding |
| ATP11B | 0.766407656 | Protein coding |
| MTF1 | 0.762988739 | Protein coding |
| CMTM6 | 0.759163639 | Protein coding |
| TACC3 | 0.758726043 | Protein coding |
| SLC12A6 | 0.757607347 | Protein coding |
| RNF149 | 0.751883181 | Protein coding |
| COP1 | 0.749381297 | Protein coding |
| LIMK2 | 0.743474641 | Protein coding |
| GRK6 | 0.74015263 | Protein coding |
| MSL3 | 0.737357459 | Protein coding |
| CTDP1 | 0.735596054 | Protein coding |
| ZNF746 | 0.735420229 | Protein coding |
| CRLF3 | 0.733430053 | Protein coding |
| ARPC2 | 0.730738917 | Protein coding |
| ANKRD13A | 0.72942198 | Protein coding |
| FAM53C | 0.729194371 | Protein coding |
| OSTF1 | 0.726730447 | Protein coding |
| MAPK14 | 0.725261852 | Protein coding |
| PFKFB4 | 0.724237804 | Protein coding |
| MYD88 | 0.719241558 | Protein coding |
| IL10RB | 0.717889834 | Protein coding |
| ARID3A | 0.716136415 | Protein coding |
| H3-3A | 0.715228636 | Protein coding |
| XPO6 | 0.72860361 | Protein coding |

**Supplementary Table 4. Top 28 co-expressed patterns of SRPK1 of differential tissue expression in “Functional Mapping and Annotation of Genome-Wide Association Studies” (FUMA)**

| Tissues/Genes | MTF1 | CAPZA1 | RNF149 | ARPC2 | CMTM6 | ATP11B | TACC3 | GRK6 | CYB5R4 | ZNF746 | USP15 | BAZ1A | SLC12A6 | PPP4C | CRLF3 | TRIM25 | CTDP1 | LIMK2 | MSL3 |
| --- | --- | --- | --- | --- | --- | --- | --- | --- | --- | --- | --- | --- | --- | --- | --- | --- | --- | --- | --- |
| Adipose Subcutaneous | 2.837795931 | 5.649724037 | 5.117336311 | 5.672425342 | 5.07847347 | 4.158047891 | 2.995345522 | 4.088284215 | 2.667729087 | 3.037805264 | 3.710909361 | 4.499344848  3.570942  3.570942745  745 | 3.570942745 | 5.670293232  3.1529323  3.152932313  13 | 3.152932313  4.951104849 | 4.951104849 | 3.546047095  4.349330667 | 4.349330667 | 4.211451385 |
| Adipose Visceral  Omentum | 2.924049885 | 5.643595043 | 5.292019991 | 5.672425342 | 5.058821599 | 3.986971781 | 3.2269554 | 4.15592924 | 2.361220726 | 2.952965418 | 3.356415056 | 4.395369667 | 3.361014794 | 5.660098391 | 3.150970686 | 4.763427907 | 3.190979265 | 4.62413484 | 3.953111909 |
| Adrenal Gland | 2.604055203 | 5.550938006 | 4.554461277 | 5.631050621 | 4.748322932 | 2.804455856 | 2.841373011 | 3.616726914 | 1.870054492 | 2.752085874 | 3.103009689 | 3.226194869 | 2.594230788 | 5.648002948 | 2.236524916 | 4.844570232 | 2.959986494 | 3.912234043 | 3.245494452 |
| Artery Aorta | 2.92534499 | 5.634352042 | 5.0847016 | 5.672425342 | 5.125519682 | 4.130805275 | 2.551274993 | 4.416427774 | 2.741419738 | 2.979466652 | 3.440671823 | 3.561192438 | 4.644407063 | 5.669015566 | 3.160809818 | 4.189330095 | 3.633671242 | 4.756122804 | 4.205894302 |
| Artery Coronary | 2.943069254 | 5.641429885 | 5.055755099 | 5.672425342 | 5.349396622 | 3.983080693 | 2.936508319 | 4.400849755 | 2.63489086 | 3.004701861 | 3.435908988 | 3.85441038 | 3.457899792 | 5.660568201 | 3.223970036 | 4.310937729 | 3.523536207 | 4.626900933 | 4.222480519 |
| Artery Tibial | 2.906510585 | 5.450094115 | 4.754904611 | 5.672425342 | 5.197490417 | 4.215891777 | 1.955231122 | 4.324187785 | 2.822991195 | 2.92395281 | 3.586134825 | 3.89315963 | 3.064263089 | 5.660880597 | 2.949676945 | 3.694346264 | 3.71050141 | 4.217755937 | 4.022428838 |
| Bladder | 2.99869147 | 5.615199354 | 5.007118567 | 5.672425342 | 5.286881614 | 4.157998452 | 2.935266577 | 4.510864356 | 2.699139797 | 3.273660607 | 3.381739 | 4.287901629 | 3.180496319 | 5.671270697 | 3.087422068 | 4.551417114 | 3.714233441 | 4.547412974 | 4.024385396 |
| Brain Amygdala | 2.054567283 | 4.563172375 | 3.147834683 | 5.30141109 | 3.160246318 | 1.66759826 | 1.415831124 | 3.305721868 | 1.248952825 | 2.018304446 | 1.483241041 | 1.951740303 | 2.478943761 | 4.520512142 | 1.952944965 | 2.602799173 | 1.856941508 | 3.953753814 | 2.90347018 |
| Brain Anterior cingulate cortex_BA241 | 1.940236152 | 4.694340998 | 3.267649251 | 5.449624297 | 2.985598898 | 1.778378616 | 1.703497013 | 3.827728138 | 1.360036951 | 2.32592546 | 1.700001024 | 1.59227535 | 2.731223061 | 4.608377433 | 2.134505237 | 2.770236364 | 1.982802255 | 3.624020354 | 3.193000292 |
| Brain Caudate basal ganglia | 2.153078755 | 4.589233375 | 3.037311567 | 5.342080348 | 3.158689142 | 1.699783231 | 1.480987777 | 3.273887195 | 1.522045662 | 2.165761706 | 1.586339706 | 1.846492348 | 2.77338378 | 4.611101074 | 2.04951892 | 2.914333645 | 2.074827638 | 4.209037299 | 2.832096227 |
| Brain Cerebellar Hemisphere | 3.186820197 | 5.347315142 | 4.358021883 | 5.562706052 | 3.409044734 | 3.431107847 | 2.844163812 | 3.84992974 | 3.290376553 | 3.542188235 | 3.245427423 | 1.865384299 | 3.838804533 | 5.582047497 | 3.826211224 | 4.339624799 | 3.131474815 | 4.842348627 | 3.958951953 |
| Brain Cerebellum | 3.208826957 | 5.013783921 | 4.155459119 | 5.574885762 | 3.323850264 | 3.405880611 | 2.798923066 | 3.955106043 | 3.088794972 | 3.599880722 | 3.237050567 | 1.877943154 | 3.742222008 | 5.608847237 | 3.54117094 | 4.477411134 | 3.210113235 | 4.971547532 | 3.938744691 |
| Brain Cortex | 2.192538185 | 4.532948608 | 3.328508627 | 5.553655409 | 2.749025551 | 1.997741419 | 1.935870796 | 4.052860532 | 1.489578486 | 2.727829199 | 1.914400243 | 1.719964904 | 2.873064471 | 4.755388801 | 2.196227489 | 3.055863028 | 2.465487901 | 3.751963052 | 3.500938002 |
| Brain Frontal Cortex BA9 | 2.217377915 | 4.992688399 | 3.626767946 | 5.564919662 | 3.037412586 | 2.128841501 | 2.024277641 | 4.016872177 | 1.65217906 | 2.718811215 | 2.059537671 | 1.679010977 | 3.146213931 | 4.880778348 | 2.48109369 | 3.001843554 | 2.338106454 | 3.572172528 | 3.630049334 |
| Brain Hippocampus | 2.14289224 | 4.54343681 | 3.257319167 | 5.344163547 | 3.011833508 | 1.917286879 | 1.556762639 | 3.419472011 | 1.325056139 | 2.184944302 | 1.599549317 | 1.965406145 | 2.607804853 | 4.501790477 | 1.97703716 | 2.413113059 | 2.022312437 | 3.519054319 | 2.891622582 |
| Brain Hypothalamus | 2.186308201 | 5.094131279 | 3.762234875 | 5.33442433 | 3.328396745 | 2.056993941 | 1.771473133 | 3.639576696 | 1.597591909 | 2.358070283 | 1.846081986 | 1.989499868 | 2.899578352 | 4.724948615 | 2.342034712 | 2.991115887 | 2.279519078 | 4.082055532 | 3.184020341 |
| Brain Nucleus accumbens basal ganglia | 2.070266692 | 4.541464817 | 3.138764876 | 5.387642581 | 2.916809897 | 1.682903249 | 1.350594068 | 3.427285434 | 1.622846275 | 2.239186903 | 1.620872586 | 1.854033645 | 2.847227152 | 4.600491495 | 2.170435968 | 3.033224071 | 2.157177299 | 4.090376793 | 2.998880185 |
| Brain Putamen basal ganglia | 2.003813416 | 4.331505208 | 2.753743133 | 5.224923143 | 2.868516329 | 1.587909854 | 1.387017523 | 3.099397361 | 1.438637465 | 1.966568296 | 1.411496994 | 1.78030511 | 2.50999374 | 4.501926648 | 1.773882076 | 2.662034143 | 2.013984378 | 3.842786746 | 2.561923127 |
| Brain Spinal cord cervical c-1 | 2.682800708 | 5.382527628 | 3.866883824 | 5.430156748 | 3.901570224 | 2.795087037 | 1.915349153 | 3.421606982 | 1.893439492 | 2.42049159 | 2.07743612 | 2.618539823 | 3.374355297 | 4.898330882 | 2.425323047 | 2.352537139 | 2.59887922 | 4.900188449 | 3.285073019 |
| Brain Substantia nigra | 2.231168819 | 4.949401646 | 3.332453225 | 5.258328638 | 3.329498969 | 2.010046831 | 1.648889244 | 3.318511627 | 1.496730505 | 1.99848828 | 1.648932489 | 2.22054253 | 2.771649757 | 4.60207497 | 2.024044168 | 2.463368173 | 2.098159833 | 4.481284285 | 2.906208824 |
| Breast Mammary Tissue | 2.771330347 | 5.637890293 | 5.030582848 | 5.672425342 | 5.114311217 | 3.962961997 | 2.740725323 | 4.231089467 | 2.426393448 | 3.020033115 | 3.438514097 | 4.422669415 | 3.350099168 | 5.668079856 | 3.123809724 | 4.655878539 | 3.51294454 | 5.013967679 | 4.098710787 |
| Cells Cultured fibroblasts | 3.642925627 | 5.672425342 | 5.398149677 | 5.672425342 | 5.670856069 | 4.690321823 | 4.489609667 | 4.385572172 | 2.639455612 | 3.280824558 | 3.358319335 | 4.999835914 | 3.338307452 | 5.672425342 | 3.491038968 | 5.570520409 | 3.538168162 | 3.50645212 | 4.064027733 |
| Cells EBV-transformed lymphocytes | 3.467247337 | 5.672425342 | 5.023350911 | 5.672425342 | 5.412629687 | 5.1304544 | 5.668558399 | 5.66558614 | 3.469579246 | 3.075310984 | 4.031642688 | 5.596329214 | 4.938957878 | 5.672425342 | 4.70925268 | 5.670174914 | 3.731641422 | 4.551981755 | 5.533341585 |
| Cervix Ectocervix | 3.155711756 | 5.656719136 | 5.308345646 | 5.672425342 | 5.171252566 | 4.582667633 | 2.852630911 | 4.277399715 | 2.435503006 | 3.339760582 | 3.463522574 | 4.331855267 | 3.911711872 | 5.659502839 | 3.626011355 | 5.025628521 | 3.566431721 | 4.929331286 | 3.980497998 |
| Cervix Endocervix | 3.136735783 | 5.66238082 | 5.139095551 | 5.672425342 | 5.37819759 | 4.303946037 | 2.909290135 | 4.38611252 | 2.434029088 | 3.599850371 | 3.378302814 | 4.402882945 | 3.446460205 | 5.672425342 | 3.434612685 | 5.141175498 | 3.65317671 | 4.97368526 | 4.181114807 |
| Colon Sigmoid | 2.865096418 | 5.515942231 | 4.537600728 | 5.672028766 | 5.005935511 | 3.988606398 | 2.764187037 | 4.511335846 | 2.418187384 | 3.094970485 | 3.164451161 | 3.783878571 | 3.060115867 | 5.661792804 | 2.804208213 | 4.208752249 | 3.471348239 | 4.353122594 | 3.950556262 |
| Colon Transverse | 2.76020145 | 5.623568311 | 4.635011396 | 5.672425342 | 5.045338106 | 3.937052665 | 3.445943702 | 4.518756461 | 2.395700349 | 2.757860094 | 3.016170886 | 3.927927011 | 2.995925645 | 5.665231318 | 2.854141059 | 4.728174114 | 3.481692173 | 5.024621595 | 3.712313251 |
| Esophagus Gastroesophageal Junction | 2.789382754 | 5.403401813 | 4.483490866 | 5.671180747 | 4.774012429 | 3.847602225 | 2.591063895 | 4.449038438 | 2.267606264 | 3.098625557 | 3.113463755 | 3.674971339 | 2.945319153 | 5.63364433 | 2.810957932 | 4.271641717 | 3.495697616 | 4.171891943 | 3.830338251 |
| Esophagus Mucosa | 3.74031904 | 5.670884392 | 5.55441804 | 5.672425342 | 5.382612762 | 5.005904451 | 4.336391798 | 4.588258834 | 2.373840234 | 2.633646294 | 3.132962532 | 4.64409573 | 4.641963592 | 5.670083039 | 3.726561982 | 4.908291961 | 3.457633391 | 5.64459286 | 3.891913141 |
| Esophagus Muscularis | 2.814471407 | 5.39422193 | 4.387258003 | 5.671924551 | 4.810939497 | 3.859504592 | 2.749281459 | 4.47980009 | 2.382436223 | 3.100831006 | 3.08336688 | 3.774894037 | 2.907377623 | 5.641315857 | 2.739972038 | 4.14315816 | 3.579258515 | 4.125851934 | 3.881811168 |
| Fallopian Tube | 3.267480715 | 5.672425342 | 5.376974103 | 5.672425342 | 5.324768493 | 4.205835723 | 2.96753555 | 4.645472345 | 2.296163809 | 3.481514166 | 3.489430546 | 4.429327365 | 3.346546664 | 5.672425342 | 3.252349915 | 5.310185352 | 3.658518185 | 5.209225987 | 4.218866808 |
| Heart Atrial Appendage | 1.689626945 | 4.499090207 | 3.759124329 | 5.553153008 | 3.686908664 | 2.858890854 | 2.066364202 | 2.926970906 | 1.528710693 | 1.896325335 | 2.477973315 | 2.485294189 | 2.377560873 | 4.823169013 | 1.820813025 | 3.200760472 | 2.174947914 | 3.180225222 | 2.681486698 |
| Heart Left Ventricle | 1.491585599 | 3.712401875 | 2.91215625 | 5.11088047 | 2.895127996 | 2.249099073 | 1.813329362 | 2.752452397 | 1.30698056 | 1.538871689 | 2.079605257 | 1.989262736 | 2.250266645 | 4.604745952 | 1.42490981 | 2.759791189 | 1.929538219 | 2.676679358 | 2.259305119 |
| Kidney Cortex | 1.898336389 | 4.827719285 | 4.000628471 | 5.472325878 | 4.042934641 | 2.582092733 | 2.166899973 | 3.137956763 | 1.639228595 | 2.205579413 | 2.18894543 | 2.609551644 | 2.693433981 | 5.312621763 | 2.00121049 | 3.50154208 | 2.784561461 | 4.287629706 | 2.961777831 |
| Kidney Medulla | 2.294382811 | 5.354849504 | 4.583647033 | 5.672425342 | 4.45891954 | 2.759540362 | 2.499350208 | 3.516338288 | 1.66548144 | 2.471876374 | 2.575309241 | 3.237127915 | 2.218953637 | 5.672425342 | 2.350464924 | 4.094441965 | 2.910126295 | 4.509218833 | 3.298202786 |
| Liver | 1.546785847 | 4.89619218 | 4.059018935 | 4.863315456 | 5.422419078 | 2.380060273 | 1.947471758 | 2.791849325 | 1.244486843 | 2.098554884 | 2.59936859 | 2.86804518 | 1.676312524 | 5.513470381 | 1.662563316 | 4.39135994 | 2.603331402 | 3.579213791 | 2.554458553 |
| Lung | 3.148324447 | 5.671931199 | 5.65625518 | 5.672425342 | 5.599986318 | 4.413616816 | 4.044529785 | 5.075393816 | 2.872358673 | 3.354186887 | 4.058007529 | 4.957916019 | 4.221147341 | 5.670890538 | 3.647979137 | 5.507805647 | 3.805957618 | 5.543848289 | 4.723883394 |
| Minor Salivary Gland | 2.661284276 | 5.501202304 | 4.861964491 | 5.661644999 | 5.22214365 | 4.140710861 | 2.745568397 | 4.290299653 | 2.265403536 | 2.702997139 | 3.02834539 | 4.242713872 | 3.233049689 | 5.59929661 | 2.878514467 | 3.996861881 | 3.354843439 | 5.516671315 | 3.734215994 |
| Muscle Skeletal | 2.419548774 | 4.235891001 | 3.049088927 | 5.666556765 | 2.037800095 | 2.645754059 | 1.325847487 | 3.258655188 | 1.905776681 | 1.824727179 | 3.072194466 | 2.963845519 | 2.788277736 | 5.313267302 | 1.397733686 | 3.344410516 | 2.629954853 | 3.300643069 | 3.593246607 |
| Nerve Tibial | 3.213682013 | 5.665760425 | 5.206078668 | 5.672425342 | 5.56384807 | 4.218485775 | 2.657220224 | 4.22068718 | 2.540549069 | 3.282439547 | 3.712823531 | 4.248588252 | 3.533283782 | 5.670297256 | 3.428244205 | 4.618474755 | 3.647886752 | 5.297475274 | 4.571025467 |
| Ovary | 3.250800349 | 5.590742785 | 5.315412633 | 5.672425342 | 4.761883467 | 4.242930102 | 2.619778838 | 4.258456121 | 2.201741424 | 3.282133324 | 3.834686619 | 4.18774774 | 3.173477465 | 5.65923009 | 3.030805767 | 4.816709289 | 2.984144092 | 4.94667152 | 3.936264532 |
| Pancreas | 1.615642459 | 4.541214199 | 4.023150118 | 4.869189154 | 3.716663771 | 2.758235458 | 1.219509424 | 2.909660932 | 1.151063683 | 1.903900969 | 2.08529527 | 2.597080579 | 1.650218482 | 4.732972897 | 1.645324549 | 3.620355039 | 2.343253267 | 3.762351731 | 2.332947364 |
| Pituitary | 3.050627438 | 5.252308252 | 4.570440547 | 5.671333047 | 4.331255628 | 3.853386303 | 2.896207397 | 4.108836432 | 2.273147668 | 3.51238699 | 3.02054502 | 2.981421585 | 3.399582374 | 5.652541514 | 3.039066646 | 4.41549379 | 3.416486928 | 4.93748811 | 3.664502381 |
| Prostate | 2.700188924 | 5.614844971 | 4.863672298 | 5.669052891 | 5.118175962 | 4.003233499 | 2.684842747 | 4.667467328 | 2.253735781 | 3.268261592 | 3.08390496 | 4.204885145 | 2.827984724 | 5.662382665 | 2.963357622 | 4.251129663 | 3.682971108 | 5.384369126 | 4.040149936 |
| Skin Not Sun Exposed Suprapubic | 3.004249565 | 5.670858034 | 5.463745692 | 5.672425342 | 5.604024927 | 4.57428434 | 3.103191666 | 4.395694084 | 2.12514342 | 3.020385198 | 3.617906666 | 4.964074694 | 3.45767955 | 5.672425342 | 3.239712483 | 5.11510109 | 4.267442946 | 5.661089885 | 3.93498615 |
| Skin Sun Exposed Lower leg | 3.065258519 | 5.670428726 | 5.487165368 | 5.672425342 | 5.609279702 | 4.608128843 | 3.037328075 | 4.442956414 | 2.267463416 | 3.06215838 | 3.689030256 | 4.833657632 | 3.49653679 | 5.67115609 | 3.35683448 | 5.091644676 | 4.513207075 | 5.659560597 | 4.004730963 |
| Small Intestine Terminal Ileum | 2.723490724 | 5.644530242 | 5.031905925 | 5.672425342 | 4.987150209 | 4.093861806 | 4.339401955 | 4.961279683 | 2.658982583 | 2.950436223 | 3.444684585 | 4.394176849 | 3.805018638 | 5.666956873 | 3.437564113 | 5.060854966 | 3.598708536 | 5.101499897 | 4.454011806 |
| Spleen | 3.035273575 | 5.672425342 | 5.663503503 | 5.672425342 | 5.512951441 | 4.009970693 | 5.410133914 | 5.669328319 | 3.187667179 | 3.89249429 | 4.381704225 | 5.166807863 | 4.349069594 | 5.672425342 | 4.40628056 | 5.645034101 | 4.095037896 | 5.47180113 | 5.413656053 |
| Stomach | 2.44583649 | 5.359840725 | 4.516249999 | 5.669782401 | 4.749227071 | 4.268864573 | 2.812480333 | 4.048407858 | 2.014905288 | 2.59819827 | 2.847547757 | 3.566155686 | 2.620835143 | 5.581348631 | 2.375529332 | 4.446937087 | 3.217703467 | 4.771376686 | 3.405441414 |
| Testis | 3.963377144 | 5.672425342 | 5.263147136 | 5.672425342 | 5.403626196 | 4.321267729 | 5.666059039 | 5.276789761 | 3.692560667 | 4.375022372 | 3.973950849 | 5.521302032 | 4.929793909 | 5.671079729 | 4.005818125 | 4.496662641 | 5.055703761 | 5.113423075 | 4.312524694 |
| Thyroid | 2.832778761 | 5.667875723 | 4.96387735 | 5.671832657 | 5.462993096 | 3.843520631 | 2.867844179 | 4.185711138 | 2.210319483 | 3.284679733 | 3.364427715 | 4.208985293 | 4.037808284 | 5.670124379 | 3.068732819 | 4.688830049 | 3.741059302 | 5.629580886 | 4.247857942 |
| Uterus | 3.235552191 | 5.651221359 | 5.117495938 | 5.672425342 | 5.208031742 | 4.445384827 | 2.758789753 | 4.725882184 | 2.522348479 | 3.528098998 | 3.443106426 | 4.121415609 | 3.615044232 | 5.670036969 | 3.325403654 | 4.941865852 | 3.738225174 | 5.013552047 | 4.235503469 |
| Vagina | 3.238187018 | 5.66954208 | 5.455329028 | 5.672425342 | 5.326099092 | 4.884181685 | 3.39763877 | 4.385420131 | 2.490721058 | 3.119575766 | 3.477866382 | 4.556165871 | 4.668825569 | 5.672425342 | 3.581455601 | 4.847095891 | 3.599981298 | 5.392964344 | 4.096547424 |
| Whole Blood | 3.520040604 | 5.52338589 | 5.624169857 | 5.66159982 | 5.262588248 | 4.388692933 | 5.464326862 | 5.642846707 | 3.712844585 | 3.918240016 | 4.439522064 | 4.681041793 | 4.642141373 | 5.637618449 | 3.724750116 | 5.409898458 | 4.103019635 | 5.551316767 | 4.933317749 |

| Tissues/Genes | PFKFB4 | FAM53C | MAPK14 | OSTF1 | ANKRD13A | XPO6 | ARID3A | IL10RB |
| --- | --- | --- | --- | --- | --- | --- | --- | --- |
| Adipose Subcutaneous | 1.339857104 | 4.227136207 | 5.146711241 | 5.670613085 | 4.578384171 | 5.338555494 | 2.834288806 | 4.474606972 |
| Adipose Visceral  Omentum | 1.483936619 | 4.37740883 | 5.013123317 | 5.653957014 | 4.110281498 | 5.178672236 | 2.492218885 | 4.511673784 |
| Adrenal Gland | 1.605100695 | 4.204899865 | 4.596271237 | 5.074884038 | 4.106680909 | 4.829863316 | 1.791802547 | 3.550596702 |
| Artery Aorta | 2.161168452 | 4.737276892 | 4.997239502 | 5.591049649 | 5.030001054 | 5.305709845 | 1.866334399 | 4.090826453 |
| Artery Coronary | 1.878851654 | 4.616191792 | 4.827431539 | 5.599631402 | 4.934419028 | 5.259437308 | 1.831720922 | 4.288642779 |
| Artery Tibial | 1.610005169 | 4.812273497 | 5.0995374 | 5.454765634 | 5.112712684 | 5.320200325 | 1.602852575 | 3.861611171 |
| Bladder | 1.904034586 | 4.50984193 | 5.314518543 | 5.359614674 | 5.187908261 | 5.287334571 | 2.38665423 | 4.20525808 |
| Brain Amygdala | 1.844563148 | 3.710209105 | 2.860175602 | 4.608250645 | 3.125127431 | 4.597781725 | 1.074109143 | 2.193824533 |
| Brain Anterior cingulate cortex_BA241 | 1.890310847 | 4.172857168 | 3.203671012 | 4.664778214 | 2.993687731 | 4.708305999 | 1.102480356 | 2.151917587 |
| Brain Caudate basal ganglia | 1.836733726 | 3.613285269 | 3.061487164 | 4.645745176 | 3.738344129 | 4.70043968 | 0.907023645 | 2.459928563 |
| Brain Cerebellar Hemisphere | 3.18332192 | 5.354673053 | 4.848383081 | 5.038145136 | 4.272602313 | 5.192540324 | 2.914374167 | 2.86309439 |
| Brain Cerebellum | 3.266153427 | 5.468743355 | 4.840600491 | 4.81683417 | 4.246853866 | 5.366342631 | 2.924033045 | 2.862714445 |
| Brain Cortex | 2.124465711 | 4.517177001 | 3.639362221 | 5.093183545 | 3.313459328 | 5.150027703 | 1.333969636 | 2.350709848 |
| Brain Frontal Cortex BA9 | 1.982899795 | 4.405028076 | 3.641863553 | 5.35902142 | 3.295904088 | 4.976261174 | 1.291107251 | 2.338386832 |
| Brain Hippocampus | 1.911732336 | 3.773004797 | 2.923804375 | 4.668401398 | 3.464258276 | 4.791429454 | 0.984630348 | 2.29578506 |
| Brain Hypothalamus | 2.407494468 | 3.962530939 | 3.198941449 | 4.569579858 | 3.326830995 | 2.2795190776 | 1.1828763670 | 2.5124491837 |
| Brain Nucleus accumbens basal ganglia | 1.761764489 | 3.709265081 | 3.199820821 | 4.605110679 | 3.632440268 | 4.783406311 | 0.93099954 | 2.451073522 |
| Brain Putamen basal ganglia | 1.732784549 | 3.295143774 | 2.815524332 | 4.578606621 | 3.617668678 | 4.582016233 | 0.827017607 | 2.317276295 |
| Brain Spinal cord cervical c-1 | 2.541293612 | 3.702450723 | 3.149903826 | 5.332335564 | 4.12671846 | 5.168708861 | 1.268611823 | 3.101848943 |
| Brain Substantia nigra | 2.072236313 | 3.475538676 | 2.772252733 | 4.866691964 | 3.441751669 | 4.822197637 | 1.081569792 | 2.479061847 |
| Breast Mammary Tissue | 1.664219001 | 4.280498085 | 4.906866934 | 5.648728981 | 4.574231834 | 5.211531656 | 2.269756667 | 4.23102529 |
| Cells Cultured fibroblasts | 2.596474896 | 5.004500044 | 5.308476773 | 5.515175696 | 5.486005972 | 5.662485275 | 3.794413618 | 5.117972306 |
| Cells EBV-transformed lymphocytes | 5.162661355 | 5.051181736 | 4.819093251 | 5.672425342 | 5.612750401 | 5.672078401 | 5.432990838 | 5.282286632 |
| Cervix Ectocervix | 2.362308624 | 4.54111479 | 5.449176472 | 5.54951441 | 5.111060146 | 5.505868778 | 2.618776917 | 4.316202402 |
| Cervix Endocervix | 2.104793013 | 4.815919231 | 5.480980739 | 5.485359697 | 4.994555547 | 5.571411561 | 2.776884237 | 4.435750142 |
| Colon Sigmoid | 1.52675894 | 4.900740709 | 4.939715092 | 4.909264106 | 4.663634791 | 5.129855643 | 2.339351923 | 3.96954138 |
| Colon Transverse | 1.939476813 | 4.127186304 | 4.713290848 | 5.32952791 | 4.579622222 | 5.045805129 | 2.214501085 | 4.426529289 |
| Esophagus Gastroesophageal Junction | 1.384963996 | 4.756692089 | 5.066133795 | 5.084571983 | 5.020766413 | 5.040204341 | 2.13218622 | 3.846512911 |
| Esophagus Mucosa | 2.95296465 | 4.091057074 | 4.945045332 | 5.669279407 | 4.982315156 | 5.209013779 | 2.226251423 | 4.436512546 |
| Esophagus Muscularis | 1.287719058 | 4.717363746 | 5.124172948 | 5.092340081 | 5.072144312 | 5.082957709 | 2.174294587 | 3.793560368 |
| Fallopian Tube | 1.925393 | 4.977096981 | 5.284142471 | 5.416687007 | 4.723841136 | 5.569295195 | 2.635543246 | 4.452463725 |
| Heart Atrial Appendage | 0.962770273 | 3.550462843 | 3.709684911 | 4.670605825 | 3.267750176 | 3.719998311 | 1.255955954 | 2.947557909 |
| Heart Left Ventricle | 0.525657823 | 3.211636726 | 3.100735103 | 3.94602535 | 2.769160391 | 3.154459544 | 1.063793772 | 2.251609745 |
| Kidney Cortex | 1.688632999 | 3.21324086 | 3.945551619 | 5.079995749 | 4.064949528 | 4.266733159 | 2.220639574 | 3.19799382 |
| Kidney Medulla | 1.974228565 | 3.577168159 | 4.300375023 | 5.424293784 | 4.456410341 | 4.638148528 | 2.633344443 | 3.7082782 |
| Liver | 0.805157197 | 2.8546929 | 4.258349979 | 4.745336435 | 3.28249245 | 4.027613765 | 1.223408803 | 3.433179106 |
| Lung | 2.870653628 | 4.774411221 | 5.260458616 | 5.671538567 | 5.329705936 | 5.625636035 | 3.324503177 | 4.868651925 |
| Minor Salivary Gland | 1.992339763 | 4.255037226 | 4.510055226 | 5.521781253 | 5.141629361 | 5.087023344 | 2.003721088 | 4.186288056 |
| Muscle Skeletal | 0.822401117 | 4.795120707 | 3.921327807 | 3.07079933 | 2.613341452 | 3.678326537 | 1.042659429 | 2.635921377 |
| Nerve Tibial | 2.36000782 | 4.579027928 | 5.283331032 | 5.661560435 | 4.848318885 | 5.662046557 | 2.278128367 | 4.538631713 |
| Ovary | 2.48193628 | 4.929503816 | 4.817897601 | 5.149550955 | 5.001331078 | 5.206387331 | 1.765592557 | 4.446339213 |
| Pancreas | 2.676116209 | 3.719555751 | 3.259244582 | 3.828123038 | 3.215206523 | 3.725759893 | 1.632473596 | 3.285253682 |
| Pituitary | 3.075501924 | 4.685614741 | 4.688532791 | 5.236335291 | 4.002137391 | 5.483572771 | 2.83519729 | 3.77684435 |
| Prostate | 2.571629533 | 4.757347772 | 4.692656587 | 5.440798796 | 4.872090632 | 5.326569967 | 2.221842812 | 4.221223345 |
| Skin Not Sun Exposed Suprapubic | 2.42396672 | 4.020843224 | 5.130379913 | 5.670724373 | 5.399362976 | 5.400478222 | 2.514267548 | 4.030540406 |
| Skin Sun Exposed Lower leg | 2.232818571 | 4.076236069 | 5.312618572 | 5.669824575 | 5.523638418 | 5.484872392 | 2.504569281 | 4.260819156 |
| Small Intestine Terminal Ileum | 3.088105581 | 4.001557215 | 4.795008118 | 5.516159023 | 4.939641076 | 5.333412699 | 2.668646908 | 4.67086554 |
| Spleen | 3.718005154 | 4.764768759 | 5.465492335 | 5.672425342 | 5.525060331 | 5.669296101 | 3.53771265 | 5.404174391 |
| Stomach | 1.216088371 | 4.272933726 | 4.339280695 | 5.036079148 | 4.21822552 | 4.708634783 | 1.77174525 | 4.08442362 |
| Testis | 5.380902813 | 5.672251369 | 3.913088346 | 5.25170121 | 5.548407352 | 5.672425342 | 4.114457364 | 2.849932865 |
| Thyroid | 1.926443414 | 4.455021168 | 5.162835991 | 5.638582809 | 5.555455752 | 5.603058967 | 2.227582378 | 4.147717677 |
| Uterus | 2.125705193 | 4.974131278 | 5.584753461 | 5.413198444 | 4.759095856 | 5.590197416 | 2.488172361 | 4.379347323 |
| Vagina | 2.852790642 | 4.370059151 | 5.313885554 | 5.654374463 | 5.113160541 | 5.423211382 | 2.649769899 | 4.442913086 |
| Whole Blood | 4.931760587 | 5.448636387 | 5.248602612 | 5.641838374 | 5.163697964 | 5.661201302 | 4.082215774 | 5.151483451 |

**Supplementary Table 5. Top 30 co-expressed patterns of SRPK1 related with different diseases including neurodevelopmental and neurodegenerative diseases found from Toppgene. (**[**https://toppgene.cchmc.org/**](https://toppgene.cchmc.org/) **)**

| **30 co-expression genes of SRPK1 related with Diseases from ToppGene** | | | | | | | |
| --- | --- | --- | --- | --- | --- | --- | --- |
| Diseases | Source | p-value | q-value Bonferroni | q-value FDR B&Y | Hit Count in Query List | Hit Count in Genome | Hit in Query List |
| INFLAMMATORY BOWEL DISEASE 25, AUTOSOMAL RECESSIVE | OMIM MedGen | 1.61 x 10^-3^ | 3.55 × 10^-2^ | 7.71 × 10^-3^ | 1 | 1 | IL10RB |
| Waldenstrom macroglobulinemia | Clinical Variations | 1.61 x 10^-3^ | 3.55 × 10^-2^ | 7.71 × 10^-3^ | 1 | 1 | MYD88 |
| Charcot-marie-tooth disease, axonal,IIa 2II | Clinical Variations | 1.61 x 10^-3^ | 3.55 × 10^-2^ | 7.71 × 10^-3^ | 1 | 1 | SLC12A6 |
| Pyogenic bacterial infections due to MyD88 deficiency | Clinical Variations | 1.61 x 10^-3^ | 3.55 × 10^-2^ | 7.71 × 10^-3^ | 1 | 1 | MYD88 |
| BASILICATA-AKHTAR SYNDROME | OMIM MedGen | 1.61 x 10^-3^ | 3.55 × 10^-2^ | 7.71 × 10^-3^ | 1 | 1 | MSL3 |
| IMMUNODEFICIENCY | OMIM MedGen | 1.61 x 10^-3^ | 3.55 × 10^-2^ | 7.71 × 10^-3^ | 1 | 1 | MYD88 |
| CONGENITAL CATARACTS, FACIAL DYSMORPHISM, AND NEUROPATHY | OMIM MedGen | 1.61 x 10^-3^ | 3.55 × 10^-2^ | 7.71 × 10^-3^ | 1 | 1 | CTDP1 |
| Congenital cataracts-facial dysmorphism-neuropathy syndrome | Clinical Variations | 1.61 x 10^-3^ | 3.55 × 10^-2^ | 7.71 × 10^-3^ | 1 | 1 | CTDP1 |
| Agenesis of the corpus callosum with peripheral neuropathy | Clinical Variations | 1.61 x 10^-3^ | 3.55 × 10^-2^ | 7.71 × 10^-3^ | 1 | 1 | SLC12A6 |
| Bryant-Li-Bhoj neurodevelopmental syndrome 1 | Clinical Variations | 1.61 x 10^-3^ | 3.55 × 10^-2^ | 7.71 × 10^-3^ | 1 | 1 | H3-3A |
| NA | OMIM MedGen | 1.61 x 10^-3^ | 3.55 × 10^-2^ | 7.71 × 10^-3^ | 1 | 1 | SLC12A6 |
| Basilicata-Akhtar syndrome | Clinical Variations | 1.61 x 10^-3^ | 3.55 × 10^-2^ | 7.71 × 10^-3^ | 1 | 1 | MSL3 |
| BRYANT-LI-BHOJ NEURODEVELOPMENTAL SYNDROME 1 | OMIM MedGen | 1.61 x 10^-3^ | 3.55 × 10^-2^ | 7.71 × 10^-3^ | 1 | 1 | H3-3A |
| MACROGLOBULINEMIA, WALDENSTROM, SUSCEPTIBILITY TO, 1 | OMIM MedGen | 1.61 x 10^-3^ | 3.55 × 10^-2^ | 7.71 × 10^-3^ | 1 | 1 | MYD88 |
| AGENESIS OF THE CORPUS CALLOSUM WITH PERIPHERAL NEUROPATHY | OMIM MedGen | 1.61 x 10^-3^ | 3.55 × 10^-2^ | 7.71 × 10^-3^ | 1 | 1 | SLC12A6 |
| Macroglobulinemia, Waldenstrom, 1 | Clinical Variations | 1.61 x 10^-3^ | 3.55 × 10^-2^ | 7.71 × 10^-3^ | 1 | 1 | MYD88 |
| Inflammatory bowel disease 25 | Clinical Variations | 1.61 x 10^-3^ | 3.55 × 10^-2^ | 7.71 × 10^-3^ | 1 | 1 | IL10RB |
| Bryant-Li-Bhoj neurodevelopmental syndrome | Clinical Variations | 3.22 × 10^-3^ | 7.09 × 10^-2^ | 1.45 × 10^-2^ | 1 | 2 | H3-3A |
| Hepatitis B virus, susceptibility to | Clinical Variations | 4.83 × 10^-3^ | 1.06 × 10^-1^ | 1.96 × 10^-2^ | 1 | 3 | IL10RB |
| NA | OMIM MedGen | 4.83 × 10^-3^ | 1.06 × 10^-1^ | 1.96 × 10^-2^ | 1 | 3 | IL10RB |
| Height | GWAS | 2.11 × 10^-2^ | 4.64 × 10^-1^ | 8.16 × 10^-2^ | 2 | 144 | TRIM25, CRLF3 |
| Amyotrophic lateral sclerosis | GWAS | 2.11 × 10^-2^ | 4.64 × 10^-1^ | 8.16 × 10^-2^ | 1 | 14 | ZNF746 |
